# RNA m6A Methylation Promotes Tumor Development and WASF3 Translation in Esophageal Squamous Cell Carcinoma

**DOI:** 10.1002/mco2.70443

**Published:** 2025-10-20

**Authors:** Qi‐Xin Shang, Wen‐Hua Huang, Yan‐Ru Feng, Yu‐Shang Yang, Wei‐Peng Hu, Yi‐Xin Liu, Yong Yuan, Ai‐Fang Ji, Long‐Qi Chen

**Affiliations:** ^1^ Department of Thoracic Surgery West China Hospital of Sichuan University Chengdu Sichuan China; ^2^ Heping Hospital Affiliated with Changzhi Medical University Changzhi China

**Keywords:** WASF3, esophageal squamous cell carcinoma, m6A methylation, METTL3, IGF2BP2, lipid nanoparticles

## Abstract

Esophageal squamous cell carcinoma (ESCC) tissues exhibit abnormal N6‐methyladenosine (m6A) modification and regulator levels, but the specific effects of this dysregulation on ESCC remain unclear. WASF3 levels were significantly elevated in ESCC tissues, and ESCC patients with high WASF3 expression had significantly worse prognosis. WASF3 suppressed the proliferation of ESCC cells and inhibited colony formation and cell cycle progression. Mechanistically, METTL3 interacted with WASF3 and mediated m6A modification of its mRNA. Insulin‐like growth factor 2 mRNA‐binding protein 2 (IGF2BP2) enhanced WASF3 translation by binding to the m6A site in its 3′ untranslated region, and highly expressed WASF3 activated the mitogen‐activated protein kinase (MAPK) signaling pathway by interacting with phosphorylated p38 (p‐p38), thereby promoting ESCC progression. Moreover, removal of the m6A modification of WASF3 mRNA inhibited WASF3 expression, ESCC cell proliferation, and abolished the ability of WASF3 to bind to p‐p38 and activate MAPK signaling. LNP small interfering RNA targeting WASF3, both alone and in combination with paclitaxel, could successfully suppress ESCC tumorigenesis. Our findings demonstrate that WASF3 plays a pivotal role in ESCC and highlight the functional significance of the METTL3/m6A/WASF3/IGF2BP2 axis in regulating ESCC progression, which could facilitate the development of novel prognostic and therapeutic targets for ESCC.

## Graphical abstract

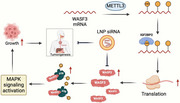

## 1 Introduction

Esophageal carcinoma is a common and deadly type of cancer, ranking sixth in terms of cancer‐related mortality globally [1, 2]. Esophageal squamous cell carcinoma (ESCC), the predominant histological ESCC subtype, accounts for approximately 90% of all ESCC cases [2, 3, 4]. While endoscopic and surgical interventions have shown promise in enhancing the 5‐year survival rate of early ESCC to 80–90% [3, 5], challenges such as atypical early symptoms, late‐stage diagnosis, limited treatment response, and high local recurrence rates continue to hinder treatment [6]. Therefore, a comprehensive understanding of the molecular mechanisms underlying ESCC progression is crucial for developing more effective interventions and improving patient outcomes.

N6‐methyladenosine (m6A), the most prevalent endogenous form of mammalian mRNA modification, involves the methylation of adenosine at the N6 position, primarily within the 3′ untranslated region (UTR) near the stop codon [7]. This modification is dynamically regulated by methyltransferases (“writers”), demethylases (“erasers”), and reader proteins (“readers”) [8, 9, 10]. Accumulating evidence suggests that m6A modification plays a pivotal role in various cellular processes, including RNA transcription, splicing, nuclear export, translation, and degradation [11, 12]. Moreover, dysregulation of m6A‐modified RNA has been implicated in numerous cancer types, highlighting its potential as a diagnostic and prognostic biomarker as well as a therapeutic target [13, 14, 15]. ESCC tissues have been previously reported to exhibit abnormal m6A RNA modification and m6A regulator levels, but the specific effects of this dysregulation on ESCC and its prognosis remained unclear.

Members of the Wiskott–Aldrich syndrome protein family play crucial roles in cell invasion and metastasis by activating the formation of membrane protrusions. Although Wiskott–Aldrich syndrome protein family member 3 (WASF3) has been shown to be essential for cancer cell motility and invasion [16, 17] as well as the formation of invasive pseudopodia [18, 19], the link between WASF3 and tumor progression is various in different cancers [20, 21], particularly in ESCC, remains unknown. Additionally, tyrosine phosphorylation of WASF3 is reportedly essential for its oncogenic activity [22], which is the only study reporting the effect of posttranslational modification of WASF3 on tumor progression [23]. However, current studies have not investigated the regulatory mechanisms underlying the aberrant expression of WASF3 in cancer, nor the specific mechanisms by which WASF3 promotes tumorigenesis, especially in ESCC.

This study was intrigued by the function of WASF3 in ESCC progression. We found WASF3 promoted ESCC tumorigenesis both in vitro and in vivo and was significantly associated with poor prognosis in ESCC patients. The abnormal expression of WASF3 in ESCC cells was dependent on the m6A modification recognized by insulin‐like growth factor 2 mRNA binding protein 2 (IGF2BP2). Furthermore, multiomics analyses identified WASF3 as a potential downstream target of METTL3 in ESCC, which mediate m6A modification of WASF3 mRNA. Further mechanistic studies revealed that mitogen‐activated protein kinase (MAPK) signaling pathway acts as a crucial downstream pathway for WASF3. WASF3 binds to phosphorylated p38 (p‐p38), which is dependent on the m6A modification of WASF3, and activate the MAPK signaling pathway subsequently. This further broadens the functional scope of WASF3. Finally, we developed and evaluated a lipid nanoparticle (LNP)‐encapsulated WASF3 small interfering RNA (siRNA) formulation for in vivo tumor therapy. Collectively, our results suggest that the METTL3/m6A/WASF3/IGF2BP2 axis could be a promising novel therapeutic target in ESCC.

## 2 Results

### 2.1 METTL3 Plays a Critical Role in ESCC Progression, and WASF3 is a Key Effector of its Function

**FIGURE 1:**
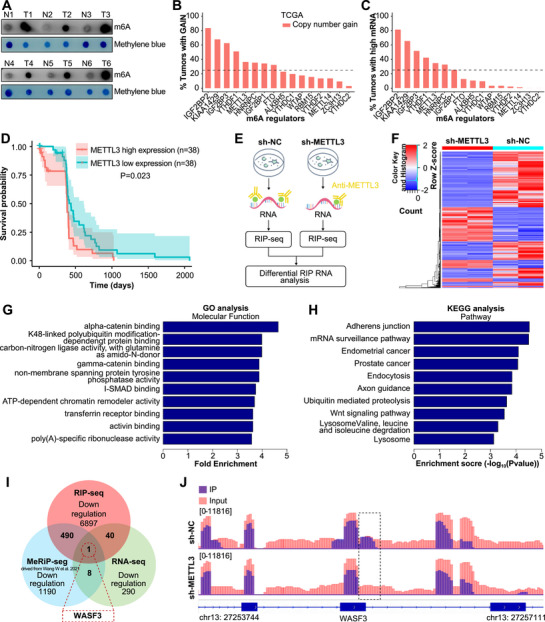
WASF3 as a potential downstream target of METTL3, identified using multiomics. (A) m6A dot blot analysis results for ESCC and adjacent tissues. (B and C) Copy number variation and mRNA expression of m6A regulators were analyzed using the TCGA database. (D) Kaplan–Meier analysis based on TCGA data. (E) Schematic diagram of RIP‐seq results. (F) Heatmap of RIP‐seq results after METTL3 knockdown. (G) GO enrichment analysis of DEGs from RIP‐seq data after METTL3 knockdown. (H) Top KEGG enrichment analysis results for DEGs from RIP‐seq data after METTL3 knockdown. (I) Venn diagram illustrating the overlap between DEGs (RNA‐seq), genes containing m6A peaks (m6A meRIP‐seq), and METTL3‐bound genes (METTL3 RIP‐seq). (J) Integrative Genomics Viewer results showing the change in the enrichment abundance of WASF3 after METTL3 knockdown.

To evaluate the potential role of m6A modifications in ESCC, we first examined m6A expression levels in ESCC and adjacent normal tissues. Dot blot analysis revealed significantly higher m6A levels in ESCC tissues than in adjacent normal tissues (Figures 1A and S1A and Table S4). Analysis of the CNVs of 16 m6A‐related regulators in the TCGA database revealed increased copy numbers of IGF2BP2, KIAA1429, IGF2BP3, YTHDF3, and METTL3 in ESCC tumors (Figure 1B); furthermore, the increase in the copy numbers of IGF2BP3 and METTL3 were consistent with the upregulation of their mRNA expression (Figure 1C). Given that METTL3 is the most important component of the methyltransferase complex and that METTL3‐mediated abnormal m6A modification is implicated in tumorigenesis, we focused on METTL3 in this study.

TCGA database results indicated that METTL3 expression was upregulated in most cancer tissues (Figure S1B), suggesting an oncogenic role of METTL3. METTL3 has significantly high expression in digestive tract tumors such as cholangiocarcinoma and liver hepatocellular carcinoma, and higher expression was associated with poorer prognosis (Figure S1C,D), suggesting that METTL3 plays an important role in ESCC. Survival analysis revealed that patients with high METTL3 expression had significantly shorter survival times compared with those with low METTL3 expression (Figure 1D). Multivariate analysis further confirmed that high METTL3 expression was an independent predictor of poor prognosis in patients with ESCC (Figure S1E). Reverse transcription quantitative polymerase chain reaction (RT‐qPCR) and western blotting confirmed that METTL3 mRNA and protein expression was significantly upregulated in esophageal cancer tissues (Figure S1F‒H). Similarly, METTL3 mRNA (Figure S1I) and protein (Figure S1J) levels were significantly higher in the nine ESCC cell lines than in normal esophageal epithelial Het‐1a cells. Based on these findings, we selected two cell lines with relatively high METTL3 expression levels (KYSE‐450 and KYSE‐150) for further investigation. These results indicate that the overall m6A modification is abnormally increased in ESCC and that METTL3‐mediated m6A modification may play a key role in cancer progression.

To dissect the mechanisms underlying METTL3‐mediated m6A modification in the regulation of ESCC progression, we constructed stable METTL3 knockdown cell lines and used RIP‐seq to identify METTL3‐bound targets (Figure 1E). RIP‐seq showed that the abundance of 10,542 transcripts was altered, with 3645 and 6897 transcripts being significantly upregulated and downregulated, respectively (Figure 1F). Gene Ontology and Kyoto Encyclopedia of Genes and Genomes analyses indicated that the enriched differentially expressed genes (DEGs) were associated with malignant tumor phenotypes (Figure 1G,H). To further clarify the genes associated with m6A modification, we compared our data with previously published methylated RNA immunoprecipitation (meRIP)‐seq and RNA‐seq data [24]. Notably, WASF3 was the only gene common to all three datasets (Figure 1I). Furthermore, visualization using an integrated genome browser revealed differences in METTL3 enrichment on WASF3 when METTL3 was knocked down (Figure 1J). Then, we detected the m6A levels of the WASF3 transcript in 12 pairs of ESCCs and adjacent normal esophageal samples and found that its m6A modification was elevated in ESCC tumors (Figure S1K and Table S4).

### 2.2 METTL3‐Mediated m6A Modification of WASF3 in ESCC

**FIGURE 2:**
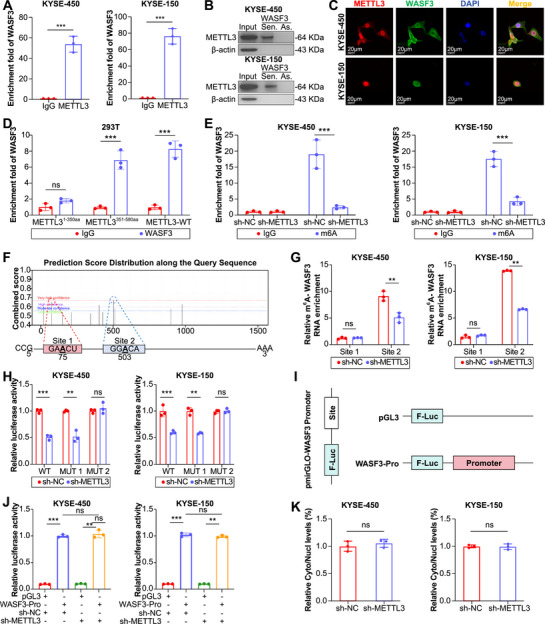
WASF3 stability is regulated by m6A modification. (A) RIP‐qPCR for detection of METTL3 binding to WASF3 mRNA in ESCC cells. (B) RNA pull‐down assay for detection of METTL3 binding to WASF3 mRNA in ESCC cells. (C) Immunofluorescence confocal microscopy to detect the colocalization of METTL3 and WASF3 in ESCC cells. (D) RIP‐qPCR detection of the binding of WASF3 to the METTL3 truncation fragment in 293T cells. (E) meRIP‐qPCR detection of m6A modification changes in WASF3 after METTL3 knockdown in ESCC cells. (F) SRAMP prediction of m6A sites on WASF3 mRNA. (G) meRIP‐qPCR analysis of m6A levels at WASF3 m6A sites in ESCC cells with or without METTL3 silencing. (H) Luciferase activities of wild‐type (wt) and mutant WASF3 3′‐UTR reporters in ESCC cells transfected with wt or mutant pmirGLO–WASF3 3′‐UTR plasmids. (I) Schematic representation of the structure of the pGL3 luciferase reporter in which the promoter region of WASF3 was cloned. (J) Dual‐luciferase reporter assay of WASF3 promoter activity in ESCC cells with or without METTL3 silencing. (K) Analysis of WASF3 expression in nuclear and cytoplasmic fractions of ESCC cells by RT‐qPCR. Data represent the mean ± SD values, *n* = 3. **p < 0.01, ***p < 0.001; ns, not significant; two‐tailed unpaired Student's *t*‐test.

To validate the RIP‐seq results, we performed RIP and RNA pull‐down experiments to determine whether WASF3 interacts with METTL3. The results showed that METTL3 and WASF3 interacted with each other (Figure 2A,B), and immunofluorescence analysis confirmed that both proteins colocalized mainly in the nucleus (Figure 2C). Therefore, we explored the specific site of interaction between METTL3 and WASF3 based on the characteristics of the METTL3 domains (Figure S2A). RIP‐qPCR results showed that the key region for the interaction between METTL3 and WASF3 was located in the C‐terminal domain (351–580 amine acids [aa]) of METTL3 (Figure 2D); this region contains the core functional methyltransferase domain of METTL3.

We then explored the role of METTL3 in ESCC. Knockdown of METTL3 significantly reduced the m6A level of WASF3 mRNA in ESCC cell lines (Figure 2E). The most common sequence motif for m6A was RRACU (R represents G or A); therefore, we used SRAMP (http://www.cuilab.cn/sramp) to predict the m6A sites in WASF3 mRNA. The results showed two high‐confidence m6A sites adjacent to the stop codon (Figure 2F). We used meRIP‐qPCR to detect these two m6A sites. m6A modification was enriched at site 2 and was significantly reduced after METTL3 knockdown (Figure 2G). Next, we constructed a dual‐luciferase reporter, which was located before the wild‐type or mutant WASF3 sequence, and the A at the m6A site was mutant to T (Figure S2B). METTL3 knockdown reduced the luciferase activity of the wild‐type WASF3 3′‐UTR reporter gene, and mutation at site 1 also led to a decrease in luciferase activity after METTL3 knockdown (Figure 2H). Overexpression of METTL3 increased luciferase activity (Figure S2C). However, neither knockdown nor overexpression of METTL3 altered the luciferase activity at site 2 (Figure S2C). To determine the effect of m6A on WASF3, we examined the effect of METTL3 knockdown on WASF3 promoter activity. First, the WASF3 promoter region was cloned into the pGL3 luciferase reporter gene (Figure 2I). WASF3 promoter activity was not affected by reduced METTL3 expression (Figure 2J), indicating that WASF3 transcription was not affected by m6A methylation. Next, we isolated RNA from the nucleus and cytoplasm of ESCC cells and found that the subcellular localization of WASF3 was not different, further indicating that the transcription of WASF3 was not affected by m6A modification (Figure 2K). These results indicate that WASF3 is a key target of METTL3‐mediated m6A modification, and that m6A modification of WASF3 does not affect its transcription.

### 2.3 m6A Modification of WASF3 mRNA Affects WASF3 Protein Expression Levels in ESCC

**FIGURE 3:**
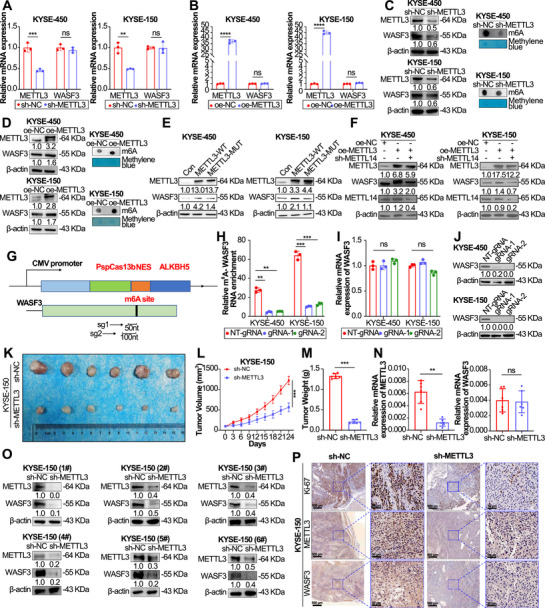
(A) m6A modification of WASF3 mRNA leads to elevated WASF3 protein levels in ESCC. RT‐qPCR analysis of METTL3 and WASF3 mRNA levels and (C) Western blotting analysis of METTL3 and WASF3 levels (left) and m6A dot blot results (right), after METTL3 knockdown in ESCC cells. (B) RT‐qPCR analysis of METTL3 and WASF3 mRNA levels after METTL3 overexpression in ESCC cells. (D) METTL3 and WASF3 levels in ESCC cells (left) and m6A dot blot results (right) after METTL3 overexpression. (E) Effects of METTL3 m6A catalytic site mutation on WASF3 levels in ESCC cells. (F) METTL3, METTL14, and WASF3 levels after transfection with the METTL3 overexpression plasmid or cotransfection with the METTL3 overexpression and METTL14 knockdown plasmids in ESCC cells. (G) Schematic representation of the dCas13b–ALKBH5 expression cassette domain organization (upper panel) and the positions of the m6A sites within the WASF3 mRNA and regions targeted by the two gRNAs (lower panel). (H) meRIP‐qPCR analysis of the WASF3 mRNA m6A levels in ESCC cells transfected with the dCas13b–ALKBH5 plasmid and control nontargeting (NT)‐gRNA or gRNA plasmid. (I) RT‐qPCR analysis of WASF3 mRNA levels in ESCC cells transfected with the dCasRX–ALKBH5 plasmid and control NT‐gRNA or gRNA plasmid. (J) Western blotting analysis of WASF3 levels in ESCC cells transfected with the dCas13b–ALKBH5 plasmid and control NT‐gRNA or gRNA plasmid. (K) KYSE‐150 cell METTL3 knockdown xenograft tumor formation experiment in nude mice. (L) Changes in subcutaneous tumor volume and (M) weight. (N) RT‐qPCR and (O) western blotting analyses of METTL3 and WASF3 expression in six pairs of subcutaneous tumor tissues. (P) IHC staining to assess Ki‐67, METTL3, and WASF3 expression in mouse subcutaneous tumor tissues. Data represent the mean ± SD, *n* = 3. **p < 0.01, ***p < 0.001, ****p < 0.000; ns, not significant; two‐tailed unpaired Student's *t*‐test.

When METTL3 expression decreased, WASF3 mRNA levels did not change significantly (Figure 3A), and overexpression of METTL3 did not change WASF3 mRNA expression (Figure 3B). However, when METTL3 expression was knocked down, both m6A and WASF3 protein levels decreased significantly, and overexpression of METTL3 significantly increased m6A and WASF3 protein levels (Figure 3C,D). FTO and ALKBH5 have been reported to function as two mammalian RNA demethylases to reverse the m6A methylation. We measured the mRNA levels of WASF3 upon ALKBH5 knockdown. The results showed that the WASF3 mRNA levels did not change significantly (Figure S2D), but the protein levels of WASF3 significantly increased after ALKBH5 knockdown (Figure S2E), and overexpression of ALKBH5 significantly decreased WASF3 protein levels (Figure S2F,G). In contrast, FTO knockdown and overexpressed did not change the mRNA and protein levels of WASF3 (Figure S2H–K). Next, we examined the effect of METTL3 m6A catalytic activity on WASF3 expression. Compared with wild‐type METTL3, transfection with the METTL3 mutant plasmid significantly reduced WASF3 protein levels in KYSE‐450 and KYSE‐150 cells (Figure 3E), indicating that the m6A catalytic activity of METTL3 is essential for regulating WASF3 expression. We also found that knockdown of METTL14, a component of the m6A methyltransferase complex, reversed the increase in WASF3 expression caused by METTL3 overexpression (Figure 3F), suggesting that WASF3 expression is coregulated by METTL3 and other methyltransferases. Using the dCas13b–ALKBH5 tool, which can demethylate targeted m6A‐modified mRNA, we designed a gRNA for the WASF3 mRNA m6A site (Figure 3G). The results showed that the expression of the two gRNAs bound to dCas13b–ALKBH5 significantly reduced the m6A level of WASF3 (Figure 3H). Next, we examined the effects of the dm6A–CRISPR system on the WASF3 mRNA and protein levels. The dm6A–CRISPR system significantly reduced WASF3 protein levels but had no significant effect on WASF3 mRNA expression (Figure 3I,J).

We also performed tumor xenograft studies to verify the regulatory effects of METTL3 on WASF3 in vivo. Knockdown of METTL3 expression significantly inhibited tumor growth compared with that in the control group (Figure 3K–M). The mRNA and protein levels of METTL3 in mouse tumor tissues were significantly decreased, whereas the mRNA expression level of WASF3 did not change significantly, even though protein expression decreased significantly (Figure 3N,O). IHC analysis revealed weaker Ki‐67 and METTL3 staining in tumors formed from ESCC cells transfected with METTL3 short hairpin RNA (shRNA) (Figure 3P). Animal experimental results showed that the effect of METTL3 knockdown on WASF3 was consistent with the results at the cellular level and in esophageal cancer tissues, affecting only its protein expression level. These results indicate that the enrichment of m6A modification of WASF3 mRNA increases its protein expression level in ESCC cells and in vivo.

### 2.4 IGF2BP2 Promotes WASF3 Protein Translation in an m6A‐Dependent Manner

**FIGURE 4:**
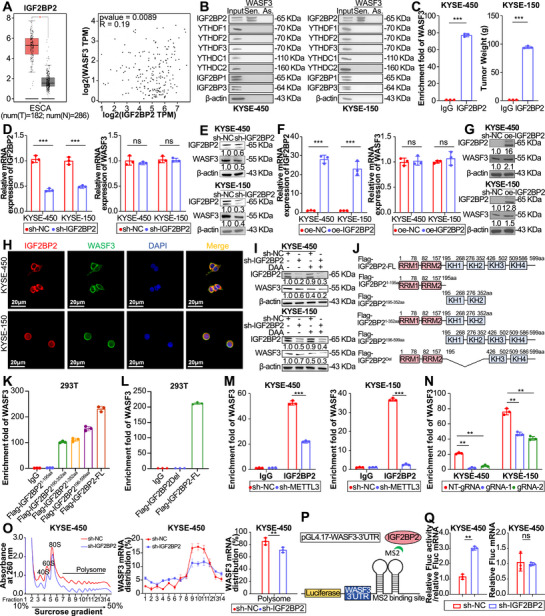
IGF2BP2 facilitates the translation of WASF3 mRNA by recognizing the m6A modification. (A) Prediction of IGF2BP2 expression and its correlation with WASF3 in ESCC using the GEPIA online database. (B) RNA pull‐down assay to detect the binding of m6A readers to WASF3 in ESCC cells. (C) RIP‐qPCR detection of IGF2BP2 binding to WASF3 mRNA in ESCC cells. (D and E) RT‐qPCR and western blotting analysis of IGF2BP2 knockdown efficiency and WASF3 expression in ESCC cells. (F and G) RT‐qPCR and western blotting analysis of IGF2BP2 overexpression efficiency and WASF3 expression in ESCC cells. (H) Immunofluorescence confocal assay to detect the colocalization of IGF2BP2 and WASF3 in ESCC cells. (I) Western blotting analysis of WASF3 expression after combined treatment with the m6A inhibitor DAA and IGF2BP2 knockdown in ESCC cells. (J) Schematic diagram of IGF2BP2 truncation and deletion fragment construction. (K) RIP‐qPCR detection of the binding of the IGF2BP2 truncation fragment to WASF3 mRNA in 293T cells. (L) RIP‐qPCR detection of IGF2BP2 KH1 and KH2 domain deletion fragment binding to WASF3 mRNA in 293T cells. (M) RIP‐qPCR detection of IGF2BP2 binding to WASF3 mRNA after METTL3 knockdown in ESCC cells. (N) RIP‐qPCR detection of the binding of IGF2BP2 to WASF3 mRNA in ESCC cells transfected with dCas13b–ALKBH5 plasmid and NT‐gRNA (control) or gRNAs plasmid. (O) Levels of WASF3 mRNA in KYSE450 cells in each gradient fraction were measured by qPCR and plotted as percentages. (P) Schematic representation of pGL4.17–WASF3–3′UTR of tethering assay. (Q) Translation efficiency and relative FLuc mRNA expression of pGL4.17–WASF3–3′UTR in KYSE450 cells. Data represent the mean ± SD values, *n* = 3. **p < 0.01, ***p < 0.001, ****p < 0.0001; ns, not significant; two‐tailed unpaired Student's *t*‐test.

To further understand how m6A methylation affects WASF3 function, we examined common m6A reader proteins (YTHDF1‐3, YTHDC1‐2, and IGF2BP1‐3) that may be involved in m6A‐mediated WASF3 dysregulation. Analyses of m6A reader protein expression and their correlations with WASF3 in ESCC using the GEPIA database revealed that only IGF2BP2 and YTHDF3 were significantly upregulated in ESCC and positively correlated with WASF3 expression (Figures 4A and S3A–G). To identify the specific m6A reader protein for WASF3, we used RNA pull‐down assays, which revealed that the sense strand probe of WASF3 mRNA could pull down IGF2BP2 (Figure 4B). This finding was further confirmed using RIP‐qPCR, which revealed WASF3 enrichment using antibodies against IGF2BP2, YTHDF1, YTHDF3, and IGF2BP3 (Figures 4C and S3H).

To further validate which m6A reader protein is involved in WASF3 mRNA m6A modification, we knocked down IGF2BP2, YTHDF1, YTHDF3, and IGF2BP3 in ESCC cells and observed that only IGF2BP2 knockdown affected WASF3 expression (Figures 4D,E and S3I). Moreover, IGF2BP2 overexpression led to a corresponding increase in WASF3 protein expression (Figure 4F,G), and immunofluorescence analysis revealed the colocalization of WASF3 and IGF2BP2 in the cytoplasm (Figure 4H). Considering these data and the finding that IGF2BP2 had the highest CNV in ESCC (Figure 1B), we concluded that IGF2BP2 recognizes the METTL3‐mediated m6A modification of WASF3 mRNA in ESCC. Considering that IGF2BP2 specifically binds to m6A‐modified transcripts, we hypothesized that IGF2BP2 regulates WASF3 expression in an m6A‐dependent manner and used 3‐deazaadenosine (DAA) to inhibit m6A modification to investigate this hypothesis. Treating KYSE‐450 and KYSE‐150 cells with DAA significantly reduced both m6A levels (Figure S3J) and WASF3 protein expression, and combined DAA treatment and IGF2BP2 knockdown further reduced WASF3 protein levels (Figure 4I).

As IGF2BP2 contains two RNA recognition motifs (RRMs) and four K‐homology (KH) domains [25], we constructed FLAG‐tagged, truncated, and full‐length IGF2BP2 fragments (Figures 4J and S3K) and used RIP‐qPCR to show that the KH1 and KH2 domains were crucial for WASF3 binding (Figure 4K,L). RIP‐qPCR also revealed that METTL3 knockdown significantly reduced the binding of IGF2BP2 to WASF3 (Figure 4M) and that the interaction between WASF3 and IGF2BP2 was significantly reduced after removing m6A modification of WASF3 using dm6A–CRISPR (Figure 4N), indicating that the m6A modification is necessary for the interaction between IGF2BP2 and WASF3 mRNA.

Knockdown or overexpression of METTL3 and IGF2BP2 expression altered only WASF3 protein, but not mRNA levels; therefore, we hypothesized that this downregulation of WASF3 expression might be due to differences in protein stability or translation efficiency. To verify this hypothesis, we added the protein synthesis inhibitor CHX to METTL3 knockdown ESCC cells. This did not result in any significant difference in the half‐life of WASF3 (Figure S4A), which indicates that the effect of METTL3 on WASF3 protein expression is not related to protein degradation.

To confirm whether IGF2BP2 promotes WASF3 translation in an m6A‐dependent manner, we used polysome profiling and confirmed that IGF2BP2 knockdown reduced the association between WASF3 transcripts and actively translated ribosomes (Figures 4O and S4B). To further investigate whether m6A methylation plays a role in the IGF2BP2‐mediated translation of WASF3 mRNA, we performed a tethering assay using a luciferase reporter plasmid containing MS2 binding sites (Figure 4P). Our results demonstrated that overexpressing IGF2BP2 in ESCC cells significantly enhanced the translation of pGL4.17–WASF3–3′UTR (Figures 4Q and S4C), indicating that IGF2BP2 promotes WASF3 translation in an m6A‐dependent manner.

### 2.5 m6A Modification of WASF3 Plays an Important Role in ESCC Progression

**FIGURE 5:**
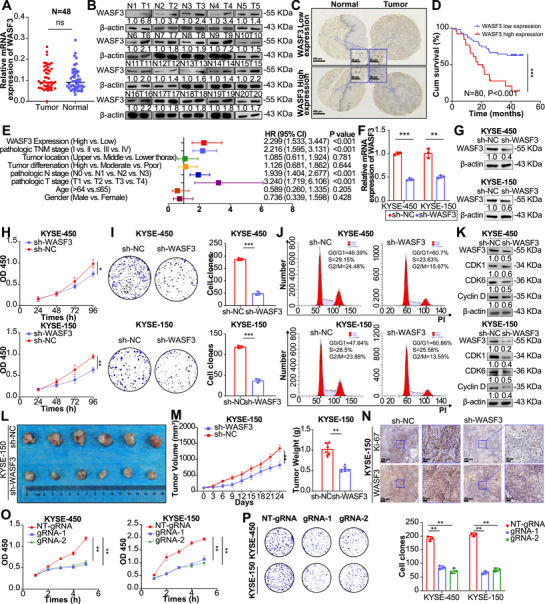
Role of WASF3 and m6A in ESCC. (A) RT‐qPCR analysis of WASF3 expression in 48 pairs of ESCC and adjacent tissues. (B) Western blotting analysis of WASF3 expression in 20 pairs of ESCC and adjacent tissues. (C) IHC staining of WASF3 in the ESCC tissue microarray. (D and E) Kaplan‒Meier analysis and multivariate Cox regression analysis based on WASF3 expression in the ESCC tissue microarray. (F and G) RT‐qPCR and western blotting analysis of WASF3 knockdown efficiency in ESCC cells. (H) CCK8 assay to detect cell proliferation ability after WASF3 knockdown in ESCC cells. (I) Colony formation assay to detect cell proliferation ability after WASF3 knockdown in ESCC cells. (J) Cell cycle assay to detect the effect of WASF3 knockdown on the cell cycle in ESCC cells. (K) Western blotting analysis of cell cycle marker changes after WASF3 knockdown in ESCC cells. (L) KYSE‐150 cell WASF3 knockdown xenograft tumor formation experiment in nude mice. (M) Changes in subcutaneous tumor volume (left) and subcutaneous tumor weight (right). (N) IHC staining to assess Ki‐67 and WASF3 expression in mouse subcutaneous tumor tissues. (O) CCK‐8 assay to detect cell proliferation in ESCC cells transfected with the dCas13b–ALKBH5 plasmid and control NT‐gRNA or gRNA plasmid. (P) Colony formation assay to assess cell proliferation ability of ESCC cells transfected with the dCas13b–ALKBH5 plasmid and control NT‐gRNA or gRNA plasmid. Data represent the mean ± SD values, *n* = 3. *p < 0.05, **p < 0.01, ***p < 0.001; ns, not significant; two‐tailed unpaired Student's *t*‐test.

To evaluate the role of WASF3 in ESCC, we examined WASF3 mRNA expression in ESCC and paired adjacent normal tissues. RT‐qPCR results revealed no difference in WASF3 mRNA levels between ESCC and adjacent normal tissues (Figure 5A). However, western blotting revealed a significant increase in WASF3 protein levels in ESCC tissues (Figure 5B). WASF3 protein expression was also significantly higher in nine ESCC cell lines than in normal esophageal epithelial Het‐1a cells (Figure S5A). Tissue microarray analysis confirmed high WASF3 expression in ESCC tumor tissues (Figure 5C), and patients with high WASF3 expression had significantly shorter survival times than those with low WASF3 expression (Figure 5D and Table S5). Multivariate analysis further demonstrated that high WASF3 expression was an independent predictor of poor prognosis in patients with ESCC (Figure 5E). To further confirm the oncogenic properties of WASF3 in ESCC, we knocked down WASF3 expression using shRNA and overexpressed WASF3 using exogenous plasmids. RT‐qPCR and western blotting confirmed knockdown and overexpression efficiencies (Figures 5F,G and S5B,C). WASF3 knockdown reduced ESCC cell proliferation and colony formation (Figure 5H,I), whereas WASF3 overexpression had the opposite effect (Figure S5D–F). Cell cycle analysis revealed that WASF3 knockdown led to cell cycle arrest at the G0/G1 phase (Figure 5J). WASF3 knockdown significantly reduced the levels of cell cycle proteins (CDK1, cyclin D1, and CDK6) (Figure 5K), whereas WASF3 overexpression promoted cell cycle progression and increased the levels of cell cycle proteins (Figure S5G,H). These results indicate that WASF3 is upregulated in ESCC and is associated with a poor prognosis. Examining the effect of WASF3 on ESCC tumor growth in vivo showed that WASF3 knockdown was found to significantly decrease tumor volume and weight compared with those in the control group (Figure 5L,M). IHC analysis revealed weaker Ki‐67 and WASF3 staining in tumors formed from ESCC cells transfected with WASF3 shRNA (Figure 5N). These results indicated that WASF3 acts as an oncogenic driver in the development and progression of ESCC.

Additionally, we found that when the m6A modification of WASF3 was removed, the proliferation and colony formation of ESCC cells were inhibited (Figure 5O,P), indicating that the m6A modification of WASF3 also affects the development of ESCC. Next, we conducted rescue experiments to further validate the role of WASF3 in METTL3‐mediated ESCC progression. We demonstrated that WASF3 overexpression rescued the proliferation and colony formation of ESCC cells with METTL3 knockdown (Figure S6A–C). Furthermore, in ESCC cells overexpressing METTL3, reducing the expression of WASF3 significantly inhibited METTL3‐induced proliferation and colony formation of ESCC cells (Figure S6D–F). These results indicate that METTL3 promotes the malignant progression of ESCC cells by upregulating the expression of WASF3.

### 2.6 m6A Modification of WASF3 is Involved in the Activation of the p38/MAPK Signaling Pathway

**FIGURE 6:**
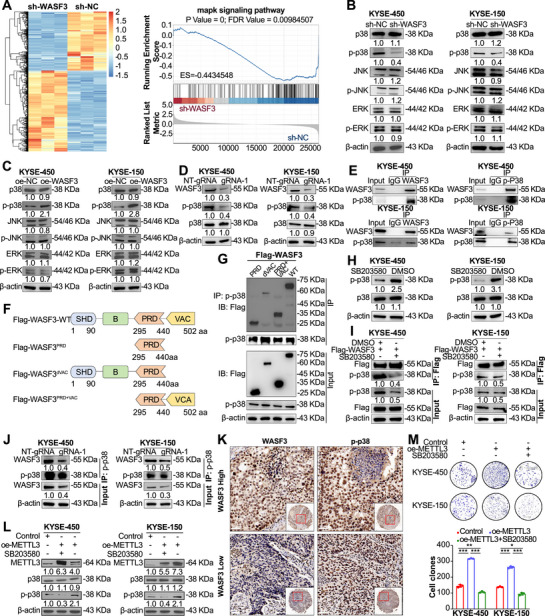
Effects of m6A methylation on WASF3‐mediated p38/MAPK signaling pathway activation. (A) Heatmap of transcriptome sequencing data after WASF3 knockdown (left). GSEA of the transcriptome sequencing data for the MAPK signaling pathway after WASF3 knockdown (right). (B) Western blotting analysis of p38, p‐p38, JNK, p‐JNK, ERK, and p‐ERK expression after WASF3 knockdown in ESCC cells. (C) Western blotting analysis of p38, p‐p38, JNK, p‐JNK, ERK, and p‐ERK expression after WASF3 overexpression in ESCC cells. (D) Western blotting analysis of p38, p‐p38, and WASF3 expression in ESCC cells transfected with the dCas13b–ALKBH5 plasmid and control NT‐gRNA or gRNA plasmid. (E) Endogenous co‐IP assay to detect the interaction between WASF3 and p‐p38 in ESCC cells. (F) Schematic diagram of WASF3 truncation fragment construction. (G) Co‐IP assay to detect the interaction between p‐p38 and WASF3 truncation fragments in 293T cells. (H) Western blotting analysis of p‐p38 expression after treatment of ESCC cells with the p38‐specific inhibitor SB203580. (I) Co‐IP assay to detect p‐p38 expression in ESCC cells after WASF3 overexpression combined with treatment with the p38‐specific inhibitor SB203580. (J) Co‐IP assay to detect the interaction between WASF3 and p‐p38 in ESCC cells transfected with the dCas13b–ALKBH5 plasmid and NT‐gRNA (control) or gRNAs plasmid. (K) IHC staining to detect the expression of WASF3 and p‐p38 in ESCC tissue microarrays. (L) Western blotting analysis of p‐p38 expression after treatment with the p38‐specific inhibitor SB203580 alone or in combination with METTL3 overexpression. (M) Colony formation assay to detect changes in cell proliferation after treatment with SB203580 alone or in combination with METTL3 overexpression. Data are shown as the mean ± SD, *n* = 3. *p < 0.05, **p < 0.01, ***p < 0.001; two‐tailed unpaired Student's *t*‐test.

To investigate the signaling pathways involved in METTL3 and WASF3 expression, we used RNA‐seq for transcriptome analysis after WASF3 knockdown and identified 739 DEGs in WASF3‐knockdown cells (312 upregulated and 427 downregulated) (Figure 6A). Gene set enrichment analysis (GSEA) revealed that the DEGs were involved in the MAPK signaling pathway (Figure 6A). To validate the RNA‐seq data, western blotting was performed, which revealed that WASF3 knockdown significantly reduced p‐p38 levels (Figure 6B), whereas WASF3 overexpression increased p‐p38 levels (Figure 6C).

Next, we investigated whether m6A modification was involved in the activation of the p38/MAPK signaling pathway mediated by WASF3. After removing the m6A modification of WASF3 mRNA, compared with control cells, the phosphorylation level of p38 was significantly reduced, and the WASF3 protein level was also decreased (Figure 6D). WASF3 acts as a scaffold protein [26]. Therefore, we hypothesized that WASF3 binds to p‐p38 to promote activation of the MAPK signaling pathway. Co‐immunoprecipitation (Co‐IP) results confirmed the interaction between WASF3 and p‐p38 (Figure 6E). To further investigate whether a specific binding domain exists between p‐p38 and WASF3, we constructed WASF3 deletion mutants based on the characteristics of their amino acid domains (Figure 6F). Co‐IP revealed that the C‐terminal domain of WASF3 interacted with p38 (Figure 6G). To prove whether the phosphorylation of p38 affects its binding to WASF3, we reduced the phosphorylation of p38 using SB203580 (a specific inhibitor of p38) (Figure 6H). Co‐IP results showed that as the phosphorylation level of p38 decreased, less WASF3 bound to it, indicating that the interaction between p38 and WASF3 depended on its phosphorylation level (Figure 6I). Next, we explored the role of m6A modification of WASF3 mRNA in the binding of p‐p38 to WASF3. The co‐IP results showed that after removing the m6A modification of WASF3 mRNA, the binding of p‐p38 to WASF3 was disrupted (Figure 6J). In addition, TMA‐based IHC confirmed that patients with high WASF3 expression exhibited increased p‐p38 levels (Figure 6K). To further validate the function of the METTL3/WASF3/p38/MAPK axis in ESCC cells, we explored the effect of SB203580, a specific inhibitor of p‐p38, on METTL3 function. The results showed that inhibition of p‐p38 expression in KYSE‐450 and KYSE‐150 cells overexpressing METTL3 eliminated the increased colony formation (Figure 6L,M). These data indicate that m6A modification of WASF3 affects the binding of WASF3 to p38 and its phosphorylation.

### 2.7 WASF3‐Targeted siRNA Delivered by LNPs Reduced Tumor Burden and Enhanced the Efficacy of Paclitaxel in ESCC

**FIGURE 7:**
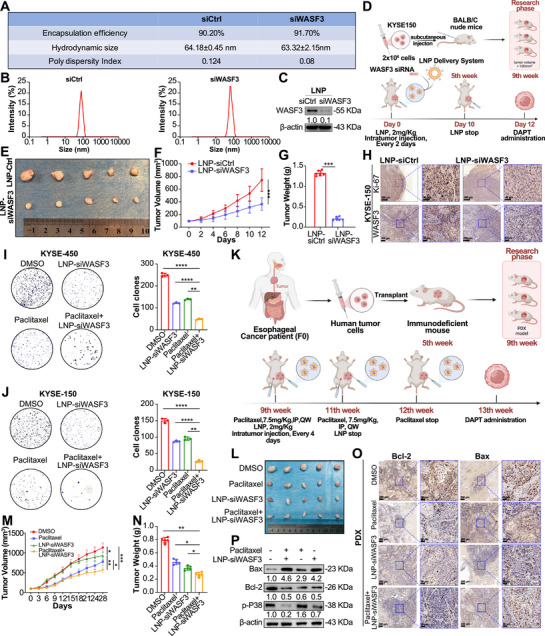
LNP siRNA targeting WASF3 for ESCC treatment in vivo. (A) Characterization of LNP siCtrl and siWASF3. (B) Dispersion of the 2′ O‐methyl‐modified LNP siCtrl and siWASF3. (C) Western blotting analysis of WASF3 protein expression in KYSE‐150 cells 48 h posttransfection with LNP siCtrl or LNP siWASF3. (D) Experimental design for LNP siWASF3 treatment in a mouse subcutaneous xenograft model. (E) Representative images of subcutaneous xenograft tumors harvested 12 days after LNP siWASF3 treatment. (F) Growth curve of subcutaneous xenograft tumors treated with LNP siWASF3. (G) Tumor weights of subcutaneous xenografts treated with LNP siWASF3. (H) IHC staining for Ki‐67 and WASF3 expression in subcutaneous xenograft tumors treated with LNP siWASF3. (I and J) Colony formation assay to detect cell proliferation after treatment of ESCC cells with LNP siWASF3 and paclitaxel alone or in combination. (K) Experimental design of PDX modeling and subcutaneous PDX treatment with LNP siWASF3 alone, paclitaxel alone, or in combination. (L) Representative images of subcutaneous PDX tumors harvested 28 days after treatment with LNP siWASF3 alone, paclitaxel alone, or both. (M) Growth curves of PDX treated with LNP siWASF3 alone, paclitaxel alone, or both. (N) Tumor weights of PDX treated with LNP siWASF3 alone, paclitaxel alone, or in combination. (O) IHC staining for Bax and Bcl‐2 expression in PDX treated with LNP siWASF3 alone, paclitaxel alone, or in combination. (P) Western blotting analysis of Bax, Bcl‐2, and p‐p38 expression in PDX treated with LNP siWASF3 alone, paclitaxel alone, or both. Data represent mean ± SD values, *n* = 3. *p < 0.05, **p < 0.01, ***p < 0.001; two‐tailed unpaired Student's *t*‐test.

Biocompatible siRNA nanoparticles have shown great potential for disease treatment; theoretically, any gene can be targeted using this approach [27]. As no drugs can currently directly target WASF3, we utilized United States Food and Drug Administration‐approved LNP technology to deliver siRNA targeting WASF3 [28]. To improve in vivo stability, all pyrimidine bases (C/U) in both strands of WASF3 siRNAs were 2′‐O‐methyl modified. Before the animal experiments, we confirmed the encapsulation efficiency, hydrodynamic size, and polydispersity index of the siRNA (Figure 7A), as well as the dispersion of the LNP (Figure 7B). In vitro knockdown experiments showed that LNP 2′‐O‐methyl modified siWASF3 effectively knocked down WASF3 gene expression at a concentration of 100 nmol/L LNP‐siRNA (Figure 7C).

According to a previous study, LNP siRNA injected into subcutaneous xenografts can distribute throughout the entire tumor tissue within hours, and the knockdown effect of a single injection can last for approximately 4 days [29]. Therefore, we subcutaneously injected ESCC tumor cells into mice and randomly divided them into two groups, receiving LNP siCtrl and LNP siWASF3 treatment, when the tumor volume reached approximately 100 mm^3^ (Figure 7D). LNP siWASF3 significantly inhibited the growth of KTSE‐150 cell xenografts (Figure 7E–G). IHC results of mouse xenograft tumor tissues showed that the expression of Ki‐67 and WASF3 in tumors decreased after LNP siWASF3 treatment (Figure 7H), indicating that LNP siWASF3 can significantly inhibit cancer cell proliferation and lead to slower xenograft growth.

Paclitaxel is a standard first‐line chemotherapeutic drug for ESCC that can significantly improve the overall survival of patients. We explored whether LNP siWASF3 could promote the efficacy of paclitaxel. Colony formation assays showed that LNP siWASF3 significantly inhibited ESCC cell proliferation in vitro and enhanced the therapeutic effect of paclitaxel (Figure 7I,J). Next, we constructed five PDX ESCC models to test the in vivo effects of LNP siWASF3 and paclitaxel (Figure 7K). Compared with the DMSO group, the LNP siWASF3 and paclitaxel groups showed significantly reduced tumor growth in the PDX models. As expected, compared with single treatments, combined treatment with LNP siWASF3 and paclitaxel had the strongest antitumor effect on PDXs (Figure 7L–N). Western blotting of subcutaneous tumor tissues from mice (Figure 7O) and IHC staining of the tumors revealed that the combination treatment group had reduced levels of cell proliferation and apoptosis inhibition markers, whereas the levels of proapoptotic markers increased (Figure 7P).

## 3 Discussion

Facing the difficulties of current treatment of ESCC and the new target with the clear and specific molecular mechanisms is warranted. The progress in next‐generation sequencing, novel systemic therapies, and immunotherapies has created substantial opportunities for the treatment of ESCC by reshaping cancer treatment paradigms and prognostic statistics. In this study, our comparison of the RIP‐seq, meRIP‐seq, and RNA‐seq data for METTL3 knockdown cells revealed that only WASF3, a scaffold protein involved in cytoskeletal organization and cell motility, was common among all three datasets. We found that WASF3 was overexpressed in ESCC tissues and cell lines through posttranscriptional m6A modification and was correlated with poor prognosis in ESCC patients. METTL3 mediated m6A modification of WASF3 mRNA, which was recognized by IGF2BP2 to promote the translation of WASF3 mRNA. Subsequently, we discovered that WASF3 interacted with p‐P38 and participated in the activation of the p38/MAPK signaling pathway, and this interaction was influenced by the m6A modification of WASF3. Finally, we demonstrated the potential of LNP siWASF3 as a therapeutic drug alone as well as in combination with paclitaxel for the treatment of ESCC.

Since our study confirmed the potential clinical value of WASF3 in ESCC, therefore, the mechanism of WASF3 upregulation in ESCC is important for exploring target points for novel treatment strategies. We found the m6A modifications contribute to the overexpression of WASF3 in ESCC. RNA undergoes extensive posttranscriptional modifications, of which m6A is the most prevalent [30]. Many studies have demonstrated the critical roles of m6A modification in various diseases [31, 32], particularly cancer [33]. We demonstrated that the upregulation of METTL3 in ESCC leads to a significant increase in m6A levels. As the dysregulation of m6A homeostasis could affect various pathophysiological processes and contribute to the development of ESCC, we then integrated multiomics data and identified WASF3 as a key downstream target of METTL3. As is known, METTL3, the first methyltransferase to be identified, is the catalytic core of the m6A methyltransferase complex, and its knockout almost completely abolishes m6A modification [34]. However, whether METTL3 mediated the m6A modification of WASF3 is the focus of our research.

METTL3, the first methyltransferase to be identified, and its knockout almost completely abolishes m6A modification [34]. In our study, METTL3 was found to regulate WASF3 expression in a catalytic activity‐dependent manner. The m6A methyltransferase complex primarily comprises METTL3, METTL14, and WTAP. METTL3 serves as the catalytic core of the methyltransferase complex, while METTL14 functions as an RNA‐binding platform [35], and both proteins cooperatively regulate gene expression. Our results show that METTL14 knockdown reversed the increased WASF3 expression induced by METTL3 overexpression, suggesting that METTL3 and METTL14 cooperatively regulate WASF3 expression. To further investigate the impact of m6A modification on WASF3 protein levels, we employed the dm6A–CRISPR system [36] to eliminate the m6A modification of WASF3 mRNA. Subsequently, we observed a significant decrease in WASF3 levels, which is consistent with previously reported findings [37]. This suggests that m6A modification of WASF3 mRNA can enhance WASF3 levels, indirectly indicating that m6A modification plays a crucial role in WASF3‐mediated oncogenic pathways.

m6A modification can regulate RNA processing, nuclear export, and translational efficiency. In this study, elevated m6A modification of WASF3 transcripts in ESCC promoted their translation. Through co‐IP to screen for readers that recognize WASF3 m6A, we found that only IGF2BP2 specifically recognizes the m6A on WASF3 mRNA. IGF2BP2 comprises two RRMs and four KH domains. IGF2BP2 can recognize m6A‐modified mRNA through its KH domains and enhance mRNA stability and translation by recruiting RNA stabilizers [11, 38]. Our results revealed that the KH1 and KH2 domains of IGF2BP2 are critical for WASF3 binding. Furthermore, by using the dm6A–CRISPR system to remove the m6A modification of WASF3 mRNA, we found that the binding ability between IGF2BP2 and WASF3 was significantly weakened. Moreover, m6A reader proteins can promote translation initiation [39, 40]. In our study, IGF2BP2 binding to the WASF3 3′UTR promoted WASF3 translation, further indicating that IGF2BP2 recognizes and promotes WASF3's translation. This may be a potential mechanism underlying the IGF2BP2‐mediated targeted mRNA regulation in ESCC.

To further explore the mechanism by which WASF3 promotes the progression of ESCC, we found that WASF3 can regulate the expression of p‐p38 and that the m6A modification of WASF3 is involved in the activation of the p‐p38/MAPK signaling pathway. p38 is an important pathway promoting cell proliferation, differentiation, migration, survival, stress responses, and apoptosis in cancer [41, 42]. Using endogenous co‐IP, we demonstrated for the first time that WASF3 binds to p‐p38 and that this interaction is dependent on the m6A modification of WASF3. This further broadens the functional scope of WASF3, currently recognized only as a scaffolding protein.

The clinical options for targeted therapy for esophageal cancer are limited. Based on our findings, targeting the METTL3/m6A/IGF2BP2/WASF3 axis could be a promising therapeutic approach. With the ability of LNP delivery systems to efficiently encapsulate siRNA and given the favorable pharmacokinetics and safety profiles, the potential for developing LNP siRNA drugs targeting the METTL3/m6A/WASF3/IGF2BP2 axis has been highlighted [28]. The effective targeted functioning of the LNP siWASF3 system was validated using an ESCC CDX model, and the synergistic effect of LNP siWASF3 and paclitaxel chemotherapy was validated using an ESCC PDX model. Therefore, targeting the METTL3/m6A/WASF3/IGF2BP2 axis with LNP siWASF3 in combination with chemotherapy, radiotherapy, or immunotherapy could be clinically beneficial, although further exploration of the feasibility and safety of such approaches is needed.

Our study has several limitations. First, we did not detect the m6A level of WASF3 mRNA in ESCC tissues. Second, our study did not focus on whether there are posttranslational modifications, such as ubiquitination and autophagy, on WASF3. Third, we did not explore the mechanism by which LNP siWASF3 promotes the efficacy of paclitaxel. Despite these limitations, we believe that the molecular mechanisms and pathways identified in our study will certainly contribute to understanding the oncogenic role of ESCC and exploring new therapeutic strategies.

## 4 Conclusions

WASF3 has high m6A modification in ESCC, and its m6A modification is mediated by METTL3 and recognized by IGF2BP2 to upregulate WASF3 translation. Highly expressed WASF3 binds to p‐p38 to activate the MAPK signaling pathway and promote ESCC progression. Given the increased m6A modification and expression of WASF3 in ESCC, as well as the pro‐oncogenic role of WASF3 m6A modification in the pathogenesis of ESCC, targeting the METTL3/m6A/WASF3/IGF2BP2 axis could be a promising therapeutic strategy to inhibit ESCC progression.

## 5 Methods

### 5.1 Clinical Samples

Tumor tissue and adjacent normal tissue (8–10 cm from the tumor boundary) were collected from patients who underwent radical esophagectomy at the West China Hospital of Sichuan University between December 2018 and January 2019. All samples were stored at −80°C until further use. All patients were informed of the study purpose and risks and provided informed consent for study participation.

### 5.2 Plasmids and shRNAs

All siRNAs and shRNAs were designed and synthesized by GenePharma (Shanghai, China) (Table S1). To generate stable knockdown cell lines, METTL3 and WASF3 shRNA were cloned and inserted into the pLV3/U6 lentiviral vector (GenePharma). IGF2BP2 shRNA, IGF2BP3–siRNA, YTH domain family, member 1 (YTHDF1)–siRNA, and YTHDF3–siRNA were purchased from GenePharma. To overexpress METTL3 and WASF3, the full‐length human gene sequences were cloned and inserted into the pCDNA3.1 expression vector. A catalytic mutant of METTL3 (aa395‐398, DPPW→APPA) was constructed based on wild‐type METTL3 and cloned and inserted into the pcDNA3.1 vector. WASF3 m6A methylation site mutants (wild‐type, A‐T) synthesized by GenePharma were cloned and inserted into the pCDNA3.1 or pmir‐GLO vectors for overexpression and dual‐luciferase assay, respectively. Cells were transfected with siRNAs or plasmids using Lipofectamine 3000 (Invitrogen) according to the manufacturer's instructions.

### 5.3 Reverse Transcription Quantitative Polymerase Chain Reaction

Total RNA was extracted using the TRIzol reagent (Invitrogen). A Cytoplasmic and Nuclear RNA Purification Kit (Norgen Biotek Corp.) was used to isolate and purify cytoplasmic and nuclear RNA from ESCC cells according to the manufacturer's protocol. Reverse transcription was performed using the PrimeScript RT reagent Kit with gDNA Eraser (TaKaRa Biotechnology). RT‐qPCR was performed using the SYBR Green PCR Master Mix (Vazyme) on an 7900HT PCR system (Applied Biosystems). GAPDH was used as an internal reference gene to normalize the mRNA levels between different samples for an exact comparison of transcription level, while 18S RNA was selected as endogenous control for the nuclear RNA. The 2‐∆∆Ct method was used to calculate relative gene expression levels. The sequences of the primers used for RT‐qPCR are listed in Table S2.

### 5.4 Western Blotting

Cells were lysed in RIPA buffer (Thermo Fisher Scientific) supplemented with protease inhibitors. After centrifugation of the samples, protein concentrations were estimated using the bicinchoninic acid method. Proteins were separated by sodium dodecyl‐sulfate polyacrylamide gel electrophoresis and transferred to polyvinylidene fluoride membranes, which were probed with primary antibodies overnight at 4°C, washed, and incubated with the appropriate secondary antibodies. Signals were visualized using chemiluminescence (Thermo Fisher Scientific). The antibodies used are listed in Table S3.

### 5.5 RNA Dot Blot Analysis

mRNA was purified using the Dynabeads mRNA Purification Kit (Thermo Fisher Scientific), diluted and spotted onto a Biodyne B membrane (Pall), and crosslinked using ultraviolet light. After blocking, the membranes were incubated with an anti‐m6A antibody. Horseradish peroxidase‐conjugated secondary antibodies and enhanced chemiluminescence reagents were used for visualization. Duplicate membranes were stained with methylene blue to ensure equal loading.

### 5.6 Co‐Immunoprecipitation

Cells were lysed in IP buffer supplemented with a protease inhibitor cocktail (CST). Proteins (200 µg) were incubated overnight with an antibody at 4°C. Protein A beads (40 µL; Thermo Fisher) were added, incubated, washed, and eluted, and the eluates were analyzed using western blotting. Cells were lysed in cell lysis buffer (CST) supplemented with a protease inhibitor cocktail (Thermo Fisher) on ice for 30 min and centrifugated at 13,000×*g* for 15 min. Cell lysates (200 µg) were incubated overnight with an antibody at 4°C. Protein A beads (40 µL; Thermo Fisher) were added and incubated with the lysates for another 4 h at 4°C. The immunoprecipitates were washed five times in Cell Lysis Buffer and boiled for 10 min in 50 µL of Blue Loading Buffer (CST).

### 5.7 Methylated RNA Immunoprecipitation qPCR

Total RNA was isolated from cells, and mRNA was purified using the NEBNext Poly(A) mRNA Magnetic Isolation Module (NEB #E7490). Magna MeRIPTM m6A Kit (Millipore) was used to measure the change of m6A levels in mRNA according to the manufacturer's protocol. mRNA (5 µg) was fragmented into 100–200 nucleotide pieces, 10% of which were saved as input, and the remaining samples were immunoprecipitated with an anti‐m6A antibody in IP binding buffer. Magna ChIP protein A/G Magnetic Beads were added, incubated, and washed, and immunoprecipitated m6A RNAs were recovered by ethanol precipitation, and the RNA concentration was measured with NanoDrop 2000.

### 5.8 Tethering Assay

The 3′UTR of WASF3 was cloned into a firefly luciferase reporter pGL4.17–firefly luciferase (FLuc)–MS2bs (Promega, USA) with MS2 binding sites. Cells were cotransfected with the relevant plasmids for 48 h, and fluorescence was then measured using the Dual‐Luciferase Reporter Assay System (E1920; Promega). Alternatively, cells were harvested for RNA extraction using TRIzol, and mRNA levels were quantified by RT‐qPCR. Firefly/Renilla ratios were calculated to determine the FLuc activity, and the relative FLuc activity was normalized to the relative FLuc–MS2bs mRNA level.

### 5.9 Statistical Analysis

Each experiment was repeated at least three times. Statistical analyses were performed using IBM SPSS version 25, GraphPad Prism version 9.1, and R version 4.3 (The R Project for Statistical Computing). Continuous variables are presented as mean and standard deviation values for normally distributed data and median and IQR values for non‐normally distributed data. Categorical variables are presented as absolute numbers and percentages. Categorical data were analyzed using the chi‐square test or Fisher's exact test. Continuous variables were analyzed using Student's *t*‐test or the Mann–Whitney *U* test. One‐way analysis of variance was used to compare three or more groups. Nonlinear regression curve fitting was performed via one‐phase decay. Survival curves were plotted using the Kaplan–Meier method, and differences between groups were analyzed using the log‐rank test. Statistical significance was set at *p* < 0.05.

Additional methods are described in Supporting Information. The data in Figure 1B, C, and D are from the official TCGA portals (https://www.cancer.gov/tcga). The data in Figures 4A and S3A–G are from other platforms (GEPIA online website: http://gepia.cancer-pku.cn). The data in Figure S1B are from other platforms (TIMER online website: http://timer.cistrome.org). The data in Figure S1C, D and F are from other platforms (UALCAN online website: https://ualcan.path.uab.edu). So neither dbGaP approval nor additional institutional clearance was required.

## Author Contributions

Yuan Y and Chen LQ provided the ESCC/adjacent normal tissues. Shang QX, Huang WH, and Feng YR designed and performed the experiments and collected ESCC/adjacent normal tissues. Huang WH and Hu WP prepared the figures and tables. Shang QX, Huang WH, and Yang YS drafted the manuscript. Shang QX and Liu YX analyzed the data. Shang QX, Huang WH, Feng YR, Yuan Y, Ji AF, and Chen LQ revised the manuscript. Yuan Y, Ji AF, and Chen LQ managed the project and approved the final version to be published. Shang QX, Yuan Y, Ji AF, and Chen LQ provided funds and resources. All authors have read and approved the final manuscript.

## Ethics Statement

The authors are accountable for all aspects of the work, ensuring that questions related to the accuracy or integrity of any part of the work are appropriately investigated and resolved. This study was conducted in accordance with the Declaration of Helsinki (as revised in 2013) and was reviewed and approved by the Human Participants’ Committee of West China Hospital of Sichuan University (approval number: No. 2021762A) and the Animal Ethics Committee of West China Hospital of Sichuan University (approval number: 20220211008). All the patients provided informed consent to participate in the study. Permission to use the resected samples and written consent were obtained preoperatively.

All experimental procedures were approved by the Institutional and Local committees on the Care and Use of Animals of West China Hospital of Sichuan University (Chengdu, China), and all the animals received human care according to the National Institutes of Health (USA) guidelines.

## Consent

All the patients provided informed consent to participate in the study. Permission to use the resected samples and written consent were obtained preoperatively.

## Conflicts of Interest

The authors declare no conflicts of interest.

## Supporting information




**Supporting Figure 1**: Expression of and prognosis based on METTL3 in TCGA database and in ESCC. m6A dot blot results of ESCC and adjacent tissues. (B) Expression of METTL3 in pan‐cancer. (C and D) Expression of and prognosis based on METTL3 in cholangiocarcinoma (CHOL) and hepatocellular carcinoma (LIHC). (E) Results of multivariate Cox regression analysis. (F) Expression of METTL3 in esophageal cancer and adjacent tissues from TCGA database. (G and H) RT‐qPCR and western blotting of 20 pairs of ESCC and adjacent tissues showing the expression of METTL3. (I and J) RT‐qPCR and western blotting analysis of METTL3 expression in nine ESCC cell lines. (K) meRIP‐qPCR analysis of m6A levels of WASF3 mRNA in ESCC tumors than in paired normal tissues (*N* = 12). Data represent mean ± SD values. *p < 0.05, **p < 0.01, ***p < 0.001; ns, not significant; two‐tailed unpaired Student's *t*‐test.
**Supporting Figure 2**: Detection of m6A modification sites on WASF3 mRNA. Schematic diagram of METTL3 truncation fragment. (B) Schematic diagram of the construction of wild‐type and m6A site mutant WASF3 dual‐luciferase reporter gene vectors. (C) Dual‐luciferase reporter assay to detect the luciferase activity of wild‐type and m6A site mutant WASF3 3′UTR reporter gene after METTL3 overexpression. (D) RT‐qPCR analysis of ALKBH5 and WASF3 mRNA levels and (E) western blotting analysis of ALKBH5 and WASF3 levels after ALKBH5 knockdown in ESCC cells. (F) RT‐qPCR analysis of ALKBH5 and WASF3 mRNA levels and (G) western blotting analysis of ALKBH5 and WASF3 levels after ALKBH5 overexpressed in ESCC cells. (H) RT‐qPCR analysis of FTO and WASF3 mRNA levels and (I) western blotting analysis of FTO and WASF3 levels after ALKBH5 knockdown in ESCC cells. (J) RT‐qPCR analysis of FTO and WASF3 mRNA levels and (K) western blotting analysis of FTO and WASF3 levels after FTO overexpressed in ESCC cells. Data represent mean ± SD values. **p < 0.01, ***p < 0.001; ns, not significant; two‐tailed unpaired Student's *t*‐test.
**Supporting Figure 3**: Validation of the correlation between m6A readers and WASF3 expression. (G) GEPIA online database analysis of the correlation between the expression of YTHDF1, YTHDF2, YTHDF3, IGF2BP1, IGF2BP3, YTHFC1, YTHDC2, and WASF3 in ESCC. (H) RIP‐qPCR detection of YTHDF1, YTHDF2, YTHDF3, IGF2BP1, IGF2BP3, YTHFC1, and YTHDC2 binding to WASF3 mRNA. (I) Western blotting analysis to detect the knockdown efficiency of YTHDF1, YTHDF3, and IGF2BP3, as well as the expression level of WASF3 in ESCC cells 48 h after transfection with YTHDF1, YTHDF3, and IGF2BP3 siRNAs. (J) m6A dot blot assay to detect the overall level of m6A after treatment with the m6A inhibitor (DAA). (K) Western blotting validation of the construction of IGF2BP2 truncation and deletion vectors. Data represent mean ± SD values. **p < 0.01, ****p < 0.0001; ns, not significance; two‐tailed unpaired Student's *t*‐test.
**Supporting Figure 4**: IGF2BP2 promotes the translation of WASF3 mRNA by recognizing m6A modifications. (A) Protein half‐life assay to detect changes in WASF3 protein expression after METTL3 knockdown. (B) Polysome profiling analysis of KYSE‐150 cell lysates after IGF2BP2 knockdown. The levels of WASF3 mRNA in each gradient fraction were measured by qPCR and plotted as a percentage in KYSE150 cells. (C) The translation efficiency and the relative FLuc mRNA expression of pGL4.17–WASF3–3′UTR in KYSE150 cells. Data represent mean ± SD values. *p < 0.05, **p < 0.01; ns, not significant; two‐tailed unpaired Student's *t*‐test.
**Supporting Figure 5**: WASF3 promotes ESCC progression. (A) Western blotting analysis of WASF3 expression in nine ESCC cell lines. (B and C) RT‐qPCR and western blotting analysis of WASF3 overexpression efficiency. (D) CCK8 assay to detect cell proliferation ability after WASF3 overexpression. (E and F) Colony formation assay to detect cell proliferation ability after WASF3 overexpression. (G) Cell cycle assay to detect the effect of WASF3 overexpression on cell cycle. (H) Western blotting analysis of cell cycle markers after WASF3 overexpression. Data represent mean ± SD values. **p < 0.01, ****p < 0.0001; two‐tailed unpaired Student's *t*‐test.
**Supporting Figure 6**: The role of WASF3 in METTL3‐mediated ESCC malignancy. (A) Western blotting analysis of METTL3, WASF3, p38, and p‐p38 levels in ESCC cells after METTL3 knockdown followed by WASF3 overexpression. (B) CCK8 assay for assessing cell proliferation after METTL3 knockdown followed by WASF3 overexpression. (C) Colony formation assay to assess changes in ESCC cell proliferation after METTL3 knockdown followed by WASF3 overexpression. (D) Western blotting analysis of METTL3, WASF3, p38, and p‐p38 levels in ESCC cells after METTL3 overexpression followed by WASF3 knockdown. (E) CCK8 assay for assessing cell proliferation after METTL3 overexpression followed by WASF3 knockdown. (F) Colony formation assay to detect changes in cell proliferation in ESCC cells after overexpression of METTL3 followed by WASF3 knockdown; data represent the mean ± SD values. *, *p* < 0.05; **, *p* < 0.01; ***, *p* < 0.001; ns, not significant; two‐tailed unpaired Student's *t*‐test.
**Supporting Table 1**: The sequences of siRNAs or shRNAs.
**Supporting Table 2**: Primers used for qRT‐PCR.
**Supporting Table 3**: List of antibodies.
**Supporting Table 4**: Characteristics of ESCC patients for dot blot and meRIP‐qPCR in this study.
**Supporting Table 5**: Baseline and clinical characteristics of patients in ESCC tissue microarray.

## Data Availability

The data associated with our study were deposited in a publicly available repository, attached to the website of the repository for accession: https://www.jianguoyun.com/p/DZfv1a8Q_9‐TDRjb9vAFIAA.
