# Supplementary material for: RNA m6A Methylation Promotes Tumor Development and WASF3 Translation in Esophageal Squamous Cell Carcinoma
Source: MedComm (2020). 2025 Oct 20;6(11):e70443. doi: 10.1002/mco2.70443 (PMC12538004; doi:10.1002/mco2.70443)
Supplement: Supplementary file 1 — Supporting Figure 1: Expression of and prognosis based on METTL3 in TCGA database and in ESCC. m6A dot blot results of ESCC and adjacent tissues. (B) Expression of METTL3 in pan‐cancer. (C and D) Expression of and prognosis based on METTL3 in cholangiocarcinoma (CHOL) and hepatocellular carcinoma (LIHC). (E) Results of multivariate Cox regression analysis. (F) Expression of METTL3 in esophageal cancer and adjacent tissues from TCGA database. (G and H) RT‐qPCR and western blotting of 20 pairs of ESCC and adjacent tissues showing the expression of METTL3. (I and J) RT‐qPCR and western blotting analysis of METTL3 expression in nine ESCC cell lines. (K) meRIP‐qPCR analysis of m6A levels of WASF3 mRNA in ESCC tumors than in paired normal tissues (N = 12). Data represent mean ± SD values. *p < 0.05, **p < 0.01, ***p < 0.001; ns, not significant; two‐tailed unpaired Student's t‐test. Supporting Figure 2: Detection of m6A modification sites on WASF3 mRNA. Schematic diagram of METTL3 truncation fragment. (B) Schematic diagram of the construction of wild‐type and m6A site mutant WASF3 dual‐luciferase reporter gene vectors. (C) Dual‐luciferase reporter assay to detect the luciferase activity of wild‐type and m6A site mutant WASF3 3′UTR reporter gene after METTL3 overexpression. (D) RT‐qPCR analysis of ALKBH5 and WASF3 mRNA levels and (E) western blotting analysis of ALKBH5 and WASF3 levels after ALKBH5 knockdown in ESCC cells. (F) RT‐qPCR analysis of ALKBH5 and WASF3 mRNA levels and (G) western blotting analysis of ALKBH5 and WASF3 levels after ALKBH5 overexpressed in ESCC cells. (H) RT‐qPCR analysis of FTO and WASF3 mRNA levels and (I) western blotting analysis of FTO and WASF3 levels after ALKBH5 knockdown in ESCC cells. (J) RT‐qPCR analysis of FTO and WASF3 mRNA levels and (K) western blotting analysis of FTO and WASF3 levels after FTO overexpressed in ESCC cells. Data represent mean ± SD values. **p < 0.01, ***p < 0.001; ns, not significant; two‐tailed unpaired Student's t [file MCO2-6-e70443-s001.docx]

**Supplementary Information**

**Title:**

RNA m6A methylation promotes tumor development and WASF3 translation in esophageal squamous cell carcinoma

**Running head:** Role of WASF3 in ESCC

Qi-Xin Shang, MD, PhD^1#^, Wen-Hua Huang, MD, PhD^1#^, Yan-Ru Feng, MD, PhD^1#^, Yu-Shang Yang, MD, PhD^1^, Wei-Peng Hu, MD, PhD^1^, Yi-Xin Liu, MD, PhD^1^, Yong Yuan, MD, PhD^1*^, Ai-Fang Ji, MD, PhD^2*^, Long-Qi Chen, MD, PhD^1*^

1 Department of Thoracic Surgery, West China Hospital of Sichuan University, Chengdu, Sichuan, China;

2 Heping Hospital Affiliated with Changzhi Medical University, No. 161 Jiefang East Street, Changzhi 046000, China.

Qi-Xin Shang^1#^, Wen-Hua Huang^1#^, and Yan-Ru Feng^1#^ contributed equally to this work.

Long-Qi Chen^1*^, Ai-Fang Ji^2*^ and Yong Yuan^1*^ are co-corresponding authors.

^1*^Co-Corresponding author. Prof. Long-Qi Chen, MD, PhD, Department of Thoracic Surgery, West China Hospital of Sichuan University, No. 37, Guoxue Alley, Chengdu, Sichuan 610041, China. Tel: +86 138 8203-0466; fax: +86 28 8542-2494; e-mail: drchenlq@scu.edu.cn

^1*^Co-Corresponding author. Prof Yong Yuan, MD, PhD, Department of Thoracic Surgery, West China Hospital of Sichuan University, No. 37, Guoxue Alley, Chengdu, Sichuan 610041, China. Tel: +86 189 8060-6739; e-mail: yongyuan@scu.edu.cn

^2*^Co-Corresponding author. Prof. Ai-Fang Ji, MD, PhD. Heping Hospital Affiliated with Changzhi Medical University, No. 161 Jiefang East Street, Changzhi 046000, China. Email: jiaifang2003@163.com.

**Figure S1**


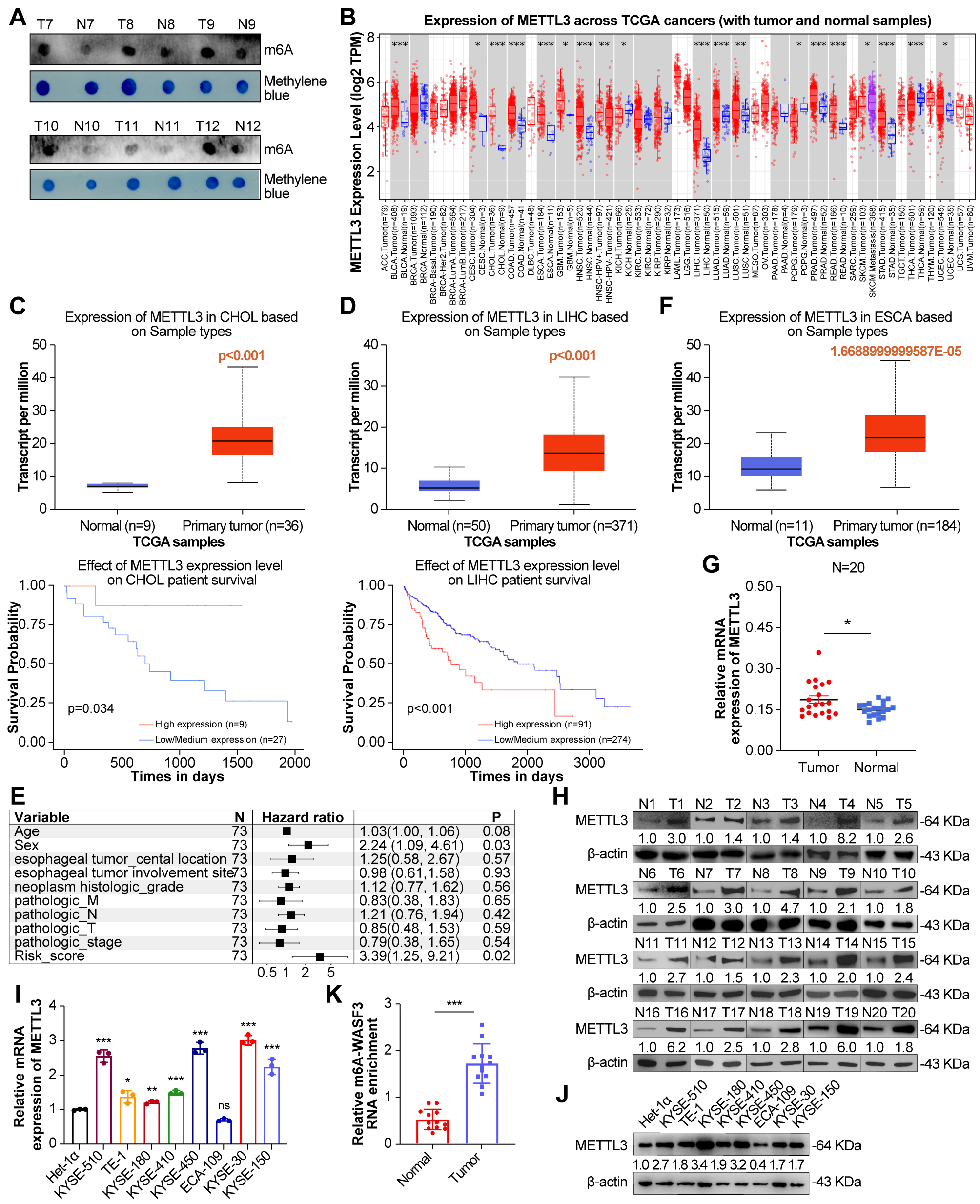


**Figure S1. Expression of and prognosis based on METTL3 in TCGA database and in ESCC.**

1. **m6A dot blot results of ESCC and adjacent tissues. (B) Expression of METTL3 in pan-cancer. (C-D) Expression of and prognosis based on METTL3 in cholangiocarcinoma (CHOL) and hepatocellular carcinoma (LIHC). (E) Results of multivariate Cox regression analysis. (F) Expression of METTL3 in esophageal cancer and adjacent tissues from TCGA database. (G-H) RT-qPCR and western blotting of 20 pairs of ESCC and adjacent tissues showing the expression of METTL3. (I-J) RT-qPCR and western blotting analysis of METTL3 expression in nine ESCC cell lines. (K) meRIP-qPCR analysis of m6A levels of WASF3 mRNA in ESCC tumors than in paired normal tissues (N = 12). Data represent mean ± SD values. * P < 0.05, ** P < 0.01, *** P < 0.001; ns, not significant; two-tailed unpaired Student’s t test.**

**Figure S2**


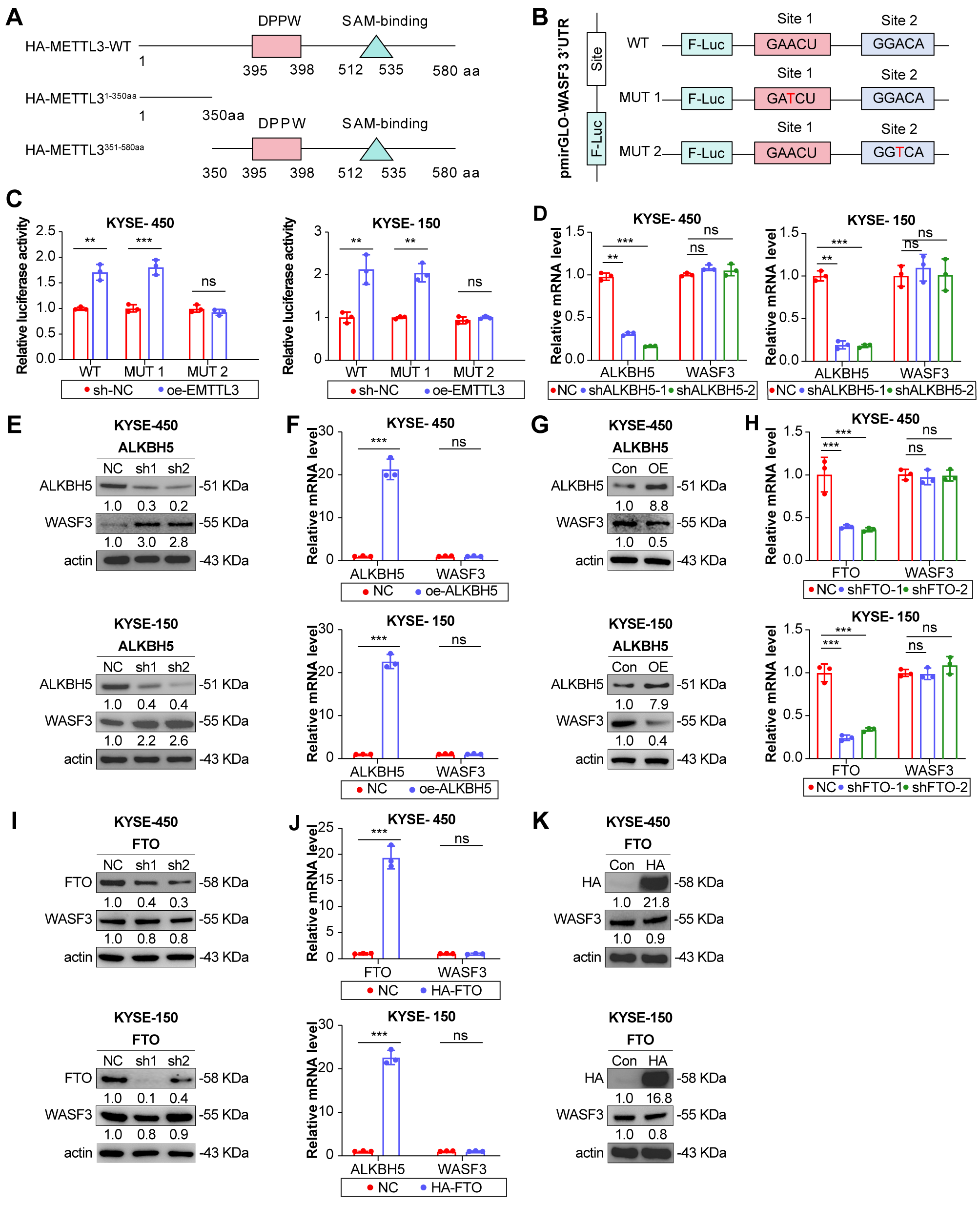


**Figure S2. Detection of m6A modification sites on WASF3 mRNA.**

1. **Schematic diagram of METTL3 truncation fragment. (B) Schematic diagram of the construction of wild-type and m6A site mutant WASF3 dual-luciferase reporter gene vectors. (C) Dual-luciferase reporter assay to detect the luciferase activity of wild-type and m6A site mutant WASF3 3'UTR reporter gene after METTL3 overexpression. (D) RT-qPCR analysis of ALKBH5 and WASF3 mRNA levels and (E) Western blotting analysis of ALKBH5 and WASF3 levels after ALKBH5 knockdown in ESCC cells. (F) RT-qPCR analysis of ALKBH5 and WASF3 mRNA levels and (G) Western blotting analysis of ALKBH5 and WASF3 levels after ALKBH5 overexpressed in ESCC cells. (H) RT-qPCR analysis of FTO and WASF3 mRNA levels and (I) Western blotting analysis of FTO and WASF3 levels after ALKBH5 knockdown in ESCC cells. (J) RT-qPCR analysis of FTO and WASF3 mRNA levels and (K) Western blotting analysis of FTO and WASF3 levels after FTO overexpressed in ESCC cells. Data represent mean ± SD values. ** P < 0.01, *** P < 0.001; ns, not significant; two-tailed unpaired Student’s t test.**

**Figure S3**

**
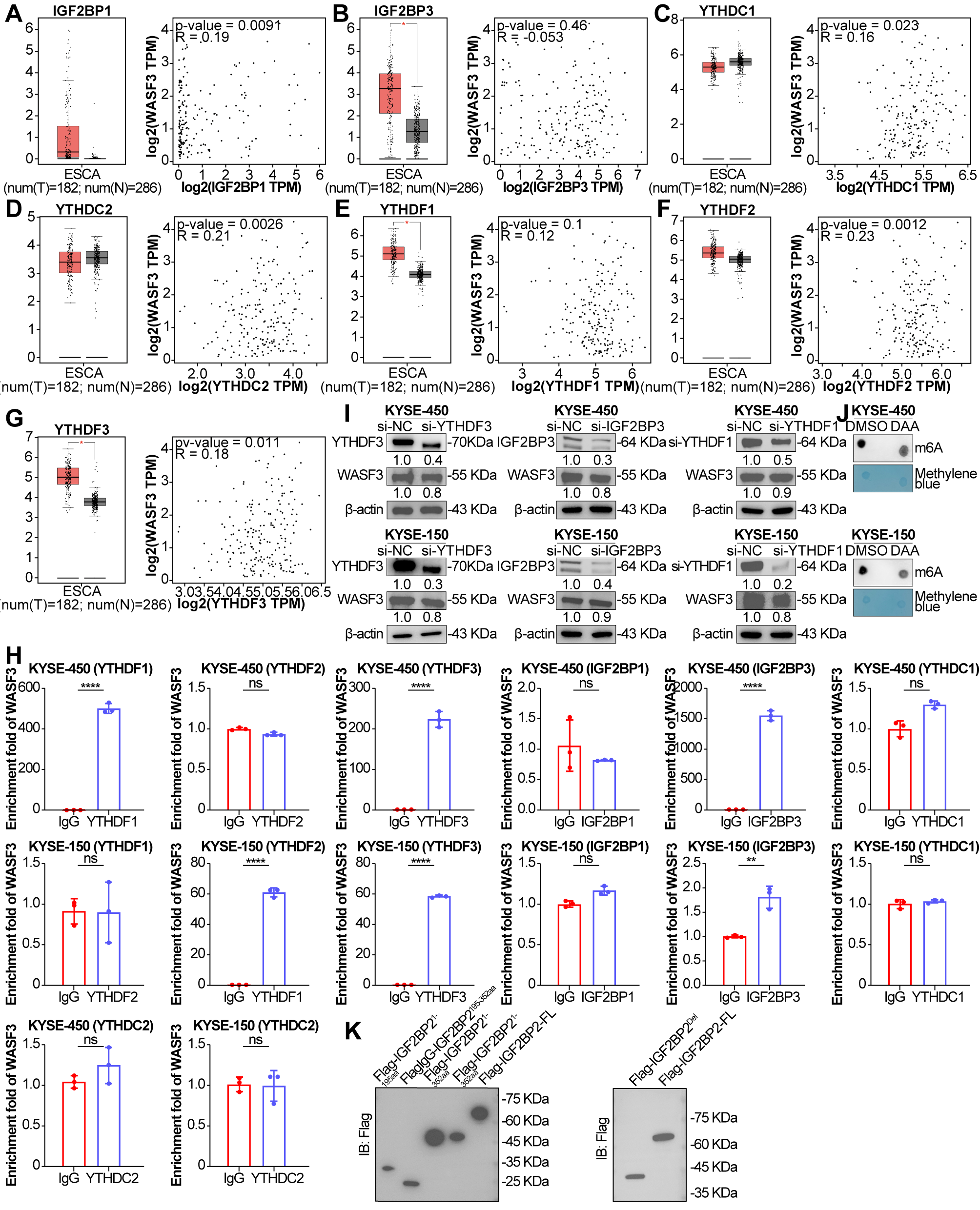
**

**Figure S3. Validation of the correlation between m6A readers and WASF3 expression.**

1. **G) GEPIA online database analysis of the correlation between the expression of YTHDF1, YTHDF2, YTHDF3, IGF2BP1, IGF2BP3, YTHFC1, YTHDC2 and WASF3 in ESCC. (H) RIP-qPCR detection of YTHDF1, YTHDF2, YTHDF3, IGF2BP1, IGF2BP3, YTHFC1, and YTHDC2 binding to WASF3 mRNA. (I) Western blotting analysis to detect the knockdown efficiency of YTHDF1, YTHDF3, and IGF2BP3, as well as the expression level of WASF3 in ESCC cells 48 h after transfection with YTHDF1, YTHDF3, and IGF2BP3 siRNAs. (J) m6A dot blot assay to detect the overall level of m6A after treatment with the m6A inhibitor (DAA). (K) Western blotting validation of the construction of IGF2BP2 truncation and deletion vectors. Data represent mean ± SD values. ** P < 0.01, **** P < 0.0001; ns, not significance; two-tailed unpaired Student’s t test.**

**Figure S4**

**
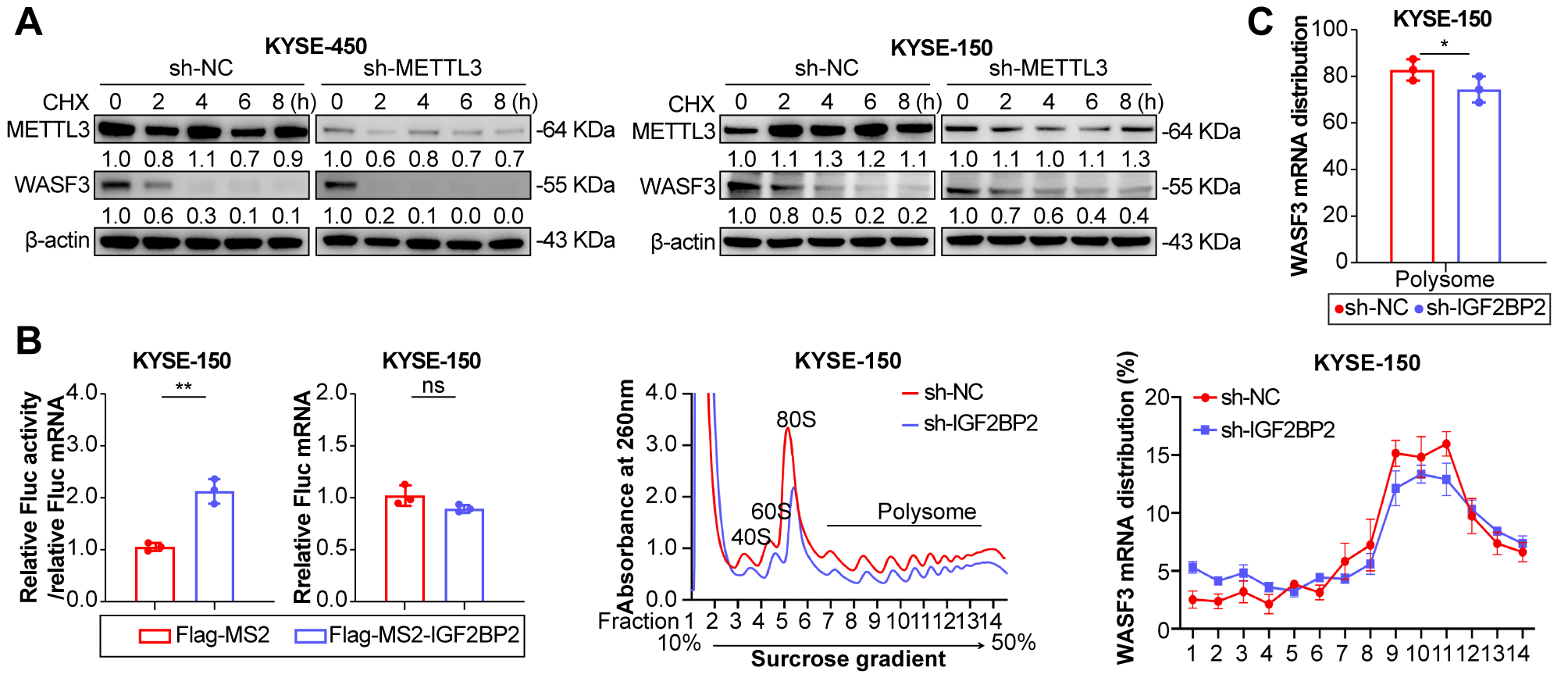
**

**Figure S4. IGF2BP2 promotes the translation of WASF3 mRNA by recognizing m6A modifications.**

**(A) Protein half-life assay to detect changes in WASF3 protein expression after METTL3 knockdown. (B) Polysome profiling analysis of KYSE-150 cell lysates after IGF2BP2 knockdown. The levels of WASF3 mRNA in each gradient fraction were measured by qPCR and plotted as a percentage in KYSE150 cells. (C) The translation efficiency and the relative FLuc mRNA expression of pGL4.17-WASF3-3′UTR in KYSE150 cells. Data represent mean ± SD values. * P < 0.05, ** P < 0.01; ns, not significant; two-tailed unpaired Student’s t test.**

**Figure S5**

**
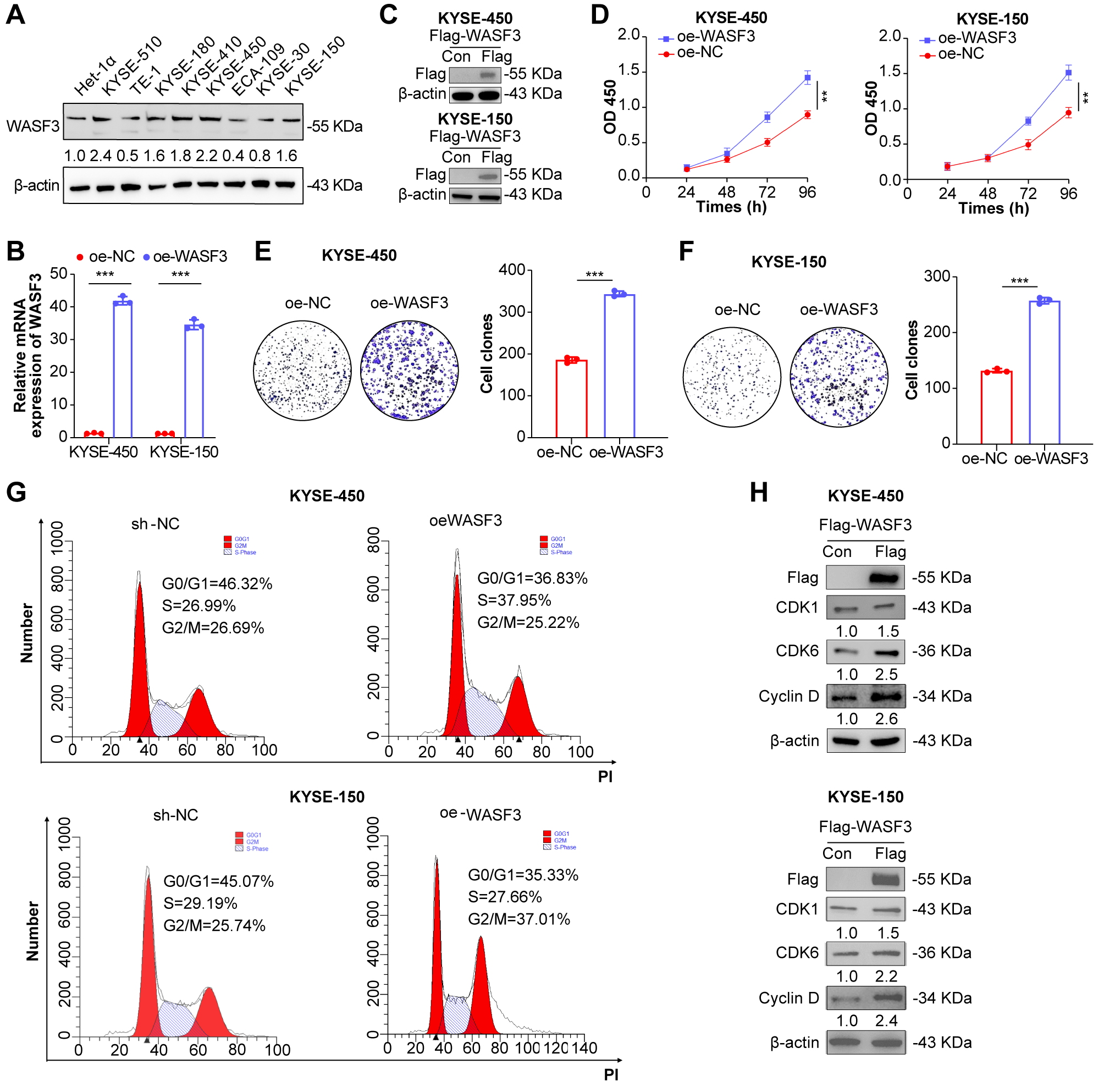
**

**Figure S5. WASF3 promotes ESCC progression.**

**(A) Western blotting analysis of WASF3 expression in nine ESCC cell lines. (B-C) RT-qPCR and western blotting analysis of WASF3 overexpression efficiency. (D) CCK8 assay to detect cell proliferation ability after WASF3 overexpression. (E-F) Colony formation assay to detect cell proliferation ability after WASF3 overexpression. (G) Cell cycle assay to detect the effect of WASF3 overexpression on cell cycle. (H) Western blotting analysis of cell cycle markers after WASF3 overexpression. Data represent mean ± SD values. ** P < 0.01, **** P < 0.0001; two-tailed unpaired Student’s t test.**

**Figure S6**

**
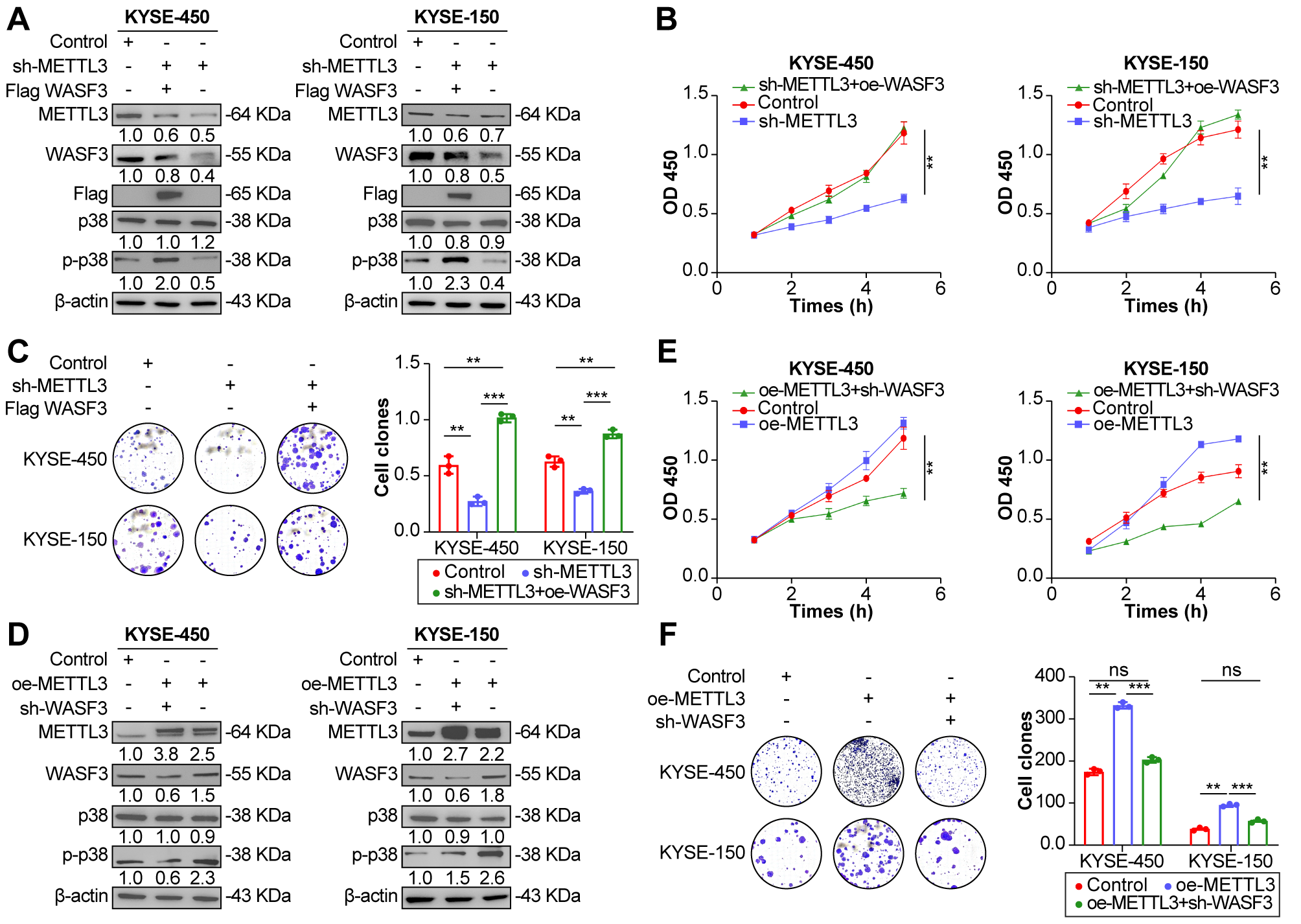
**

**Figure S6. The role of WASF3 in METTL3-mediated ESCC malignancy.**

**(A) Western blotting analysis of METTL3, WASF3, p38, and p-p38 levels in ESCC cells after METTL3 knockdown followed by WASF3 overexpression. (B) CCK8 assay for assessing cell proliferation after METTL3 knockdown followed by WASF3 overexpression. (C) Colony formation assay to assess changes in ESCC cell proliferation after METTL3 knockdown followed by WASF3 overexpression. (D) Western blotting analysis of METTL3, WASF3, p38, and p-p38 levels in ESCC cells after METTL3 overexpression followed by WASF3 knockdown. (E) CCK8 assay for assessing cell proliferation after METTL3 overexpression followed by WASF3 knockdown. (F) Colony formation assay to detect changes in cell proliferation in ESCC cells after overexpression of METTL3 followed by WASF3 knockdown; data represent the mean ± SD values. *, P < 0.05; **, P < 0.01; ***, P < 0.001; ns, not significant; two-tailed unpaired Student’s t test.**

**Table S1.** The sequences of siRNAs or shRNAs.

| shRNAs | Sequences (5’-3’) |
| --- | --- |
| METTL3-shRNA | GCUGCACUUCAGACGAAUUTT |
| YTHDF1-siRNA | CCGCGTCTAGTTGTTCATGAA |
| YTHDF3-siRNA | GCAATACAGTAGATTTGAATACCTT |
| IGF2BP3-siRNA | GCTGAGAAGTCGATTACTA |
| IGF2BP2-shRNA | AGTGAAGCTGGAAGCGCATAT |
| METTL14-shRNA | CCATGTACTTACAAGCCGATA |
| ALKBH5-shRNA#1 | CCTCAGGAAGACAAGATTA |
| ALKBH5-shRNA#2 | AGAAGGGCCTGTACAACGA |
| FTO-shRNA#1 | CCCATTAGGTGCCCATATTTA |
| FTO-shRNA#2 | TCACCAAGGAGACTGCTATTT |
| WASF3-shRNA | CCGGCGCTGCTATTCGAATGGGAATCTCGAGATTCCCATTCGAATAGCAGCGTTTTTG |
| METTL3-mut(D395A) | GCTGACCCA mutated to GCTGCCCCA |
| NC-shRNA | CCACATGAAGCAGCACGACTT |
| siControl | UUCUCCGAACGUGUCACGUTT |
| WASF3-sense | >NM_006646.6:246-1754 *Homo sapiens* WASP family member 3 (WASF3), transcript variant 1, mRNA |
| WASF3-anti-snese | >NM_006646.6:246-1754, complementary sequence of transcript variant 1 |

**Table S2.** Primers used for qRT-PCR.

| Primer names | Sequences (5’-3’) |
| --- | --- |
| For qRT‒PCR |  |
| METTL3 F | CTTCAGCAGTTCCTGAATTAGC |
| METTL3 R | ATGTTAAGGCCAGATCAGAGAG |
| METTL14 F | TGCAGCACCTCGATCATTTATTT |
| METTL14 R | AAGTCTTAGTCTTCCCAGGATTGTT |
| WASF3 F | AAAGTCACCCAGCTGGATTC |
| WASF3 R | GTGGCTTATCACTCTGGTTGTA |
| IGF2BP2 F | CATCATCGGAAAGGAGGGCTTGAC |
| IGF2BP2 R | GCATGGATGGTGACAGGCTTCTC |
| YTHDF1 F | TGTGGAATGAGGGACCGTTG |
| YTHDF1 R | GGATCCTCTACAAGGGCACG |
| YTHDF2 F | ACTTCTCAGCATGGGGAAATAA |
| YTHDF2 R | TATTCATGCCAGGAGCCTTATT |
| YTHDF3 F | GCTCCACCAACCCAACCAGTTC |
| YTHDF3 R | CTGAGGTCCTTGTTGCTGCTGTG |
| YTHDC1 F | AAAGGAAAGTCAGCCACAGAGT |
| YTHDC1 R | GAGGCACTACTTGATAGACGAA |
| YTHDC2 F  YTHDC2 R | ATGCCTGTTCGATACTTCATAATG |
|  | CACTCCACCTTAAATACTCCTCCT |
| IGF2BP1 F | GCAAGTGAACACCGAGAGTGAGAC |
| IGF2BP1 R | TGCCAATGAGACGCCCTACAAAG |
| IGF2BP3 F | TCACTTCTATGCTTGCCAGGTTGC |
| IGF2BP3 R | CCTTCTGTTGTTGGTGCTGCTTTAC |
| GADPH F | CATGTTCCAATATGATTCCACC |
| GADPH R | CACTTGATTTTGGAGGGATCTC |
| ALKBH5 F | ATCCTCAGGAAGACAAGATTAG |
| ALKBH5 R | TTCTCTTCCTTGTCCATCTC |
| FTO F |  |
| FTO R |  |
| For MeRIP | Sequences (5’-3’) |
| WASF3 F | GTGTTGAACAAACAGGTGCTCC |
| WASF3 R | CCGTCACCTCAAACATCTCTG |

**Table S3.** List of antibodies.

| Antibody name | Source | Item number |
| --- | --- | --- |
| m6A Rabbit mAb | Sigma | SAB5600251 |
| METTL3 | Abcam | EPR18810 |
| CDK1 | ABclonal | A11420 |
| CDK6 | ABclonal | A0106 |
| Cyclin D | Wanleibio | WL01435a |
| WASF3 | Proteintech | 67620-1-Ig |
| Ki-67 | Proteintech | 27309-1-AP |
| Flag | ABclonal | AE092 |
| HA | ABclonal | AE008 |
| Myc | Proteintech | 16286-1-AP |
| His | ABclonal | AE003 |
| METTL14 | ABclonal | A8530 |
| IGF2BP2 | Proteintech | 11601-1-AP |
| YTHDF1 | Abcam | ab220162 |
| YTHDF2 | Abcam | ab220163 |
| YTHDF3 | Abcam | ab220161 |
| YTHDC1 | Abcam | ab264375 |
| YTHDC2 | Abcam | ab271139 |
| IGF2BP1 | Abcam | ab184305 |
| IGF2BP3 | Abcam | ab177477 |
| P38 | CST | #9219 |
| p-P38 | CST | #9211 |
| JNK | CST | #9252 |
| p-JNK | CST | #9251 |
| ERK | CST | #9102 |
| p-ERK | CST | #9101 |
| Bax | Wanleibio | WL01637 |
| Bcl-2 | Wanleibio | WL01556 |
| GAPDH | ABclonal | A19056 |
| β-actin (Rabbit) | ABclonal | AC026 |
| β-actin (Mouse) | ABclonal | AC004 |
| Rabbit IgG | CST | #2729 |
| Mouse IgG | CST | #68860 |
| Cy3 Goat Anti-Rabbit IgG (H+L) | ABclonal | AS007 |
| HRP mouse antibody | ABclonal | AS003 |
| HRP rabbit antibody | ABclonal | AS014 |
| IHC mouse antibody | ABclonal | A17437 |
| IHC rabbit antibody | ABclonal | A23073 |
| ALKBH5 | Proteintech | 16837-1-AP |
| FTO | CST | #45980 |

**Table S4.** Characteristics of ESCC patients for dot blot and merip-qPCR in this study

| Sample ID | Gender | Age | Differentiation | TNM stage^a^ | Family  history |
| --- | --- | --- | --- | --- | --- |
| 0001176333 | Male | 78 | Moderate | III | No |
| 0032516547 | Male | 64 | Moderate | II | No |
| 0015512066 | Male | 76 | Moderate | III | No |
| 0016894712 | Female | 61 | Low | IV | No |
| 0033155843 | Male | 65 | Moderate | III | No |
| 0015011846 | Male | 62 | Moderate | III | Yes |
| 0016566379 | Female | 68 | Moderate | III | No |
| 0033280339 | Male | 68 | Low | III | No |
| 0033412928 | Male | 66 | Moderate | III | No |
| 0002956335 | Female | 72 | Moderate | III | No |
| 0000650852 | Male | 67 | Low | III | No |
| 0033632469 | Female | 69 | Moderate | II | Yes |

1. The stage were defined according to the 8th edition of the AJCC cancer Staging System.

**Table S5.** Baseline and clinical characteristics of patients in ESCC tissue microarray

| Clinicopathological Characters | | All cases (%)  80 | WASF3 expression | | P valueb |
| --- | --- | --- | --- | --- | --- |
|  |  |  | High (%) | Low (%) |  |
|  |  |  | 25 | 55 |  |
| Gender |  |  |  |  | 0.239 |
|  | Male | 63 (78.8) | 18 (72.0) | 45 (81.8) |  |
|  | Female | 17 (21.2) | 7 (28.0) | 10 (18.2) |  |
| Age，mean (S.E.M） |  | 61.5±7.8 | 61.3±8.2 | 61.8±6.6 | 0.176 |
| T stagea |  |  |  |  | <0.001 |
|  | T1 | 10 (12.5) | 5 (20.0) | 5 (9.2) |  |
|  | T2 | 10 (12.5) | 0 (0.0) | 10 (18.1) |  |
|  | T3 | 59 (73.7) | 0 (0.0) | 39 (70.9) |  |
|  | T4 | 1 (1.3) | 20 (80.0) | 1 (1.8) |  |
| N stagea |  |  |  |  | 0.050 |
|  | N0 | 46 (57.4) | 12 (48.0) | 34 (61.8) |  |
|  | N1 | 22 (27.5) | 6 (24.0) | 16 (29.2) |  |
|  | N2 | 7 (8.7) | 4 (16.0) | 3 (5.4) |  |
|  | N3 | 5 (6.3) | 3 (12.0) | 2 (3.6) |  |
| Differentiation |  |  |  |  | 0.178 |
|  | High | 10 (12.5) | 5 (20.0) | 5 (9.2) |  |
|  | Moderate | 41 (51.2) | 12 (48.0) | 29 (52.7) |  |
|  | Low | 29 (36.3) | 8 (32.0) | 21 (38.1) |  |
| Tumor location |  |  |  |  | 0.370 |
|  | Upper thoracic | 9 (11.3) | 4 (16.0) | 5 (9.2) |  |
|  | Middle thoracic | 47 (58.7) | 10 (40.0) | 37 (67.2) |  |
|  | Lower thoacic | 24 (30.0) | 11 (44.0) | 13 (23.6) |  |
| TNM stagea |  |  |  |  | 0.001 |
|  | I | 10 (12.5) | 4 (16.0) | 6 (10.9) |  |
|  | II | 38 (47.5) | 9 (36.0) | 29 (52.8) |  |
|  | III | 26 (32.5) | 9 (36.0) | 17 (30.9) |  |
|  | IV | 6 (7.5) | 3 (12.0) | 3 (5.4) |  |

1. The stage were defined according to the 8th edition of the AJCC cancer Staging System.

b. P value was calculated by two-side Chi-square test/t student test/Fisher’s exact test.

**Supplementary Methods**

**The Cancer Genome Atlas (TCGA) database analysis**

We queried the TCGA ([https://cancergenome.nih.gov](https://cancergenome.nih.gov))**-ESCA dataset (184 tumors and 11 normal tissues) and analyzed them for** copy number variations (**CNVs) and the mRNA expression of m6A regulators to validate METTL3 expression and assess its prognostic value. METTL3 expression and prognosis in other cancers were assessed using the TCGA Pan-Cancer database.**

**Tissue microarray analysis**

The protein expression of WASF3 and p-p38 in 80 cases of ESCC cancer patients by use of tissue microarray (AF-EsoSur2201, BIOTECH). The slides were processed, blocked, and incubated with antibodies targeting WASF3 and p-p38 in a humidified chamber at 4℃ overnight, followed by incubation with HRP-conjugated secondary antibody for 30 minutes at room temperature, the signal was developed with diaminobenzidine (DAB) solution. Images were analyzed using the Aperio ImageScope (Leica). The intensity was scored on a scale of 0–3 as negative (0), weak (1), medium (2), or strong (3). The extent of the staining, defined as the percentage of positive stained areas of tumor cells per the whole-tumor area, was scored on a scale of 0 (0%), 1 (1–25%), 2 (26–50%), 3 (51–75%), and 4 (76–100%). An overall protein expression score (overall score range, 0–12) was calculated by multiplying the intensity and positive scores. For statistical purposes, the staining score was further categorized as low (0–5), medium (6–8), and high (9–12).

**Cell Culture**

ESCC cell lines (KYSE-30, KYSE-410, KYSE-450, KYSE-180, TE-1, ECA-109, KYSE-150, KYSE-510), normal esophageal epithelial Het-1ɑ cells, and 293T cells were used in this study. The KYSE-30, KYSE-410, KYSE-450, KYSE-180, TE-1, ECA-109, KYSE-150, KYSE-510 cell line was a gift from Dr. Yong Yuan (Department of Thoracic Surgery, West China Hospital of Sichuan University, Chengdu, China). The 293T cell line was a gift from Dr. Gang Yuan

(Department of Thoracic Surgery, West China Hospital of Sichuan University, Chengdu, China). All cell lines were confirmed to be free of mycoplasma. The identities of the cell lines were authenticated with short tandem repeat (STR) profiling. All cell lines were cultured in 1640 medium (Gibco) supplemented with 10% fetal bovine serum (FBS) (Gibco), and all cells were incubated at 37 ℃ in a humidified atmosphere containing 5% CO_2_.

**Colony formation and Cell Counting Kit-8 (CCK-8) assays**

For the CCK-8 assay, 1000 cells in the logarithmic growth phase were seeded in 96-well plates. In brief, 20 ul of CCK-8 in 1640 medium (200 µl) was added to each well, and the cells were incubated for 2 hours at 37℃. Then, absorbance was measured at 450nm on a microplate reader (Bio-Rad). The cell growth rate was examined at 24h, 48h, 72h and 96h after seeding, and statistical results were obtained from three independent experiments. For the colony formation assay, 1000 cells were seeded in 12-well plates and cultured for 7days. The colonies were fixed with paraformaldehyde, stained with 0.2% crystal violet, and counted. Statistical results were obtained from three independent experiments.

**Cell cycle analysis**

Prepare a cell suspension (5×10^5^ cells/ml) in a 1.5 ml microcentrifuge tube. Centrifuge at 300 ×g for 5 minutes and discard the supernatant. Add 1 ml of a 70% ethanol to a microcentrifuge tube and incubate at -20°C overnight. Add 500 µl of PBS to the microcentrifuge tube, suspend by pipetting, centrifuge at 300× g for 5 minutes. The cells were re-suspended in PBS (500 µl) and 5 µl of C549 (Cell Cycle Assay Solution Blue) was added. Each tube was incubated at 37℃ for 15 minutes, protected from light. The stained cells were passed through a cell strainer and analyze samples using a flow cytometer.

**RNA sequencing (RNA-seq) and data analysis**

RNA from WASF3-knockdown cells was isolated using TRIzol (Thermo Fisher, 15596018). RNA quality and integrity were assessed using a NanoDrop ND-1000 (NanoDrop) and Bioanalyzer 2100 (Agilent), respectively. Samples with concentrations >50 ng/μL, RIN values >7.0, and total RNA >1 μg were used for downstream experiments. Poly(A) mRNA was enriched, fragmented, and reverse transcribed for double-stranded cDNA synthesis. Libraries were prepared and sequenced using the Illumina NovaSeq 6000 (LC Bio Technology Co., Ltd.).

**RNA immunoprecipitation sequencing (RIP-seq)**

RIP was performed using an anti-METTL3 antibody (Abcam, ab220162) and the EZ Magna RIP RNA-Binding Protein Immunoprecipitation Kit (Sigma, 17-701). Briefly, cell lysates were incubated with antibody-conjugated beads and washed. Thereafter, proteins were removed using proteinase K digestion, and RNA was isolated and subjected to RT-qPCR and sequencing.

**RNA pull-down assay**

Cells were lysed (1×10^7^ in 500 μL IP buffer, protease inhibitor, phosphatase inhibitor, and RNase inhibitor cocktail), and WASF3 mRNA was biotinylated using the Pierce Magnetic RNA‒Protein Pull-Down Kit (Thermo Fisher Scientific, 20164). Biotinylated mRNA was incubated with cell lysates from KYSE450 and KYSE150 cells, pulled down using magnetic beads, washed twice (with 50 mM Tris, pH 7.4, 150 mM NaCl, 0.05% NP-40, and 1 mM MgCl_2_), and eluted for western blotting.

**Polysome profiling**

Cells were treated with cycloheximide (CHX) to arrest translation and lysed (in 5 mM Tris-HCl, 2.5 mM MgCl2, 1.5 mM KCl, protease inhibitor cocktail, 5 mL of 10 mg/mL CHX, 1 mL of 1 M DTT, and 100 U RNase inhibitor). The lysate was incubated on ice for 10 min and centrifuged at 16,000 rpm for 7 min at 4 ℃ after the addition of 25 mL 10% Triton X-100 and 25 mL 10% sodium deoxycholate, followed by vortexing for 10 s. 10% of the lysate was used to determine cytosolic steady-state RNA levels. Then, the lysate was layered onto a sucrose gradient (5–50%) and centrifuged at 35,000 rpm for 2 h at 4 ℃ (Beckman). The gradient was fractionated using an ISCO fractionator, and RNA absorbance at 260 nm was monitored. Collected RNA analyzed by RT-qPCR.

**Luciferase reporter assay**

To validate the binding of METTL3 to the WASF3 m6A site, wild-type and mutant WASF3 mRNA fragments containing the m6A site were synthesized, cloned, and inserted into the pGL3 luciferase reporter vector. To validate the YY1 binding site in the METTL3 promoter, mutations were introduced into the binding sites predicted using the JASPAR website; the sequences were subsequently cloned and inserted into the pGL3 vector. After transfection, luciferase activity was measured using the Luc-Pair Duo-Luciferase HS Assay Kit (Gene Copoeia). Luciferase activity was normalized to Renilla luciferase activity.

**Immunofluorescence**

Cells were cultured on coverslips, fixed, permeabilized, blocked, and incubated with primary antibodies against METTL3, IGF2BP2, or WASF3, followed by incubation with secondary fluorescently-labeled antibodies. Nuclei were stained using DAPI. Images were captured using an inverted fluorescence microscope (Olympus).

**In vivo tumorigenesis**

All female BALB/c nude mice (3–4 weeks old) used in our study were purchased from the Beijing HFK Bioscience Co., Ltd. (China). For xenograft experiments, 2×10^6^ cells with stable METTL3 or WASF3 knockdown or negative control (NC, sh-NC), were subcutaneously injected into the right flank of each mouse. Tumor growth was monitored weekly, and tumor volume was calculated using the following formula: volume = length × width2 × 0.5. The early endpoint was defined as a tumor volume of 1500 mm3.

For the PDX models (PDX#1–5), the tumor tissues were obtained from patients receiving surgeries at our cancer center. Tumor tissues surgically resected from patients with ESCC were stored in ice-cold Dulbecco's modified Eagle’s medium (DMEM; Gibco) supplemented with 10% fetal bovine serum and antibiotics, cut into 2–3 mm3 pieces, and implanted subcutaneously into the flanks of each mouse. When the tumor volume reached 1–2 cm3, tissues were harvested from PDX tumor-bearing mice, cut into pieces, and implanted subcutaneously into to establish the PDX model. Twenty female PDX model mice were randomly assigned into the following 4 groups: control, paclitaxel (injected into the abdominal cavity, 7.5mg/kg per week), LNP-siWASF3 (injected directly into the tumor bodies, 2mg/kg every four days), or combined treatment. Tumor volume was measured every 3 days. After treatment for 4 weeks, the mice were sacrificed and all the tumors were extracted and weighed. All animal experiments were approved by the Animal Care Committee of West China Hospital, Sichuan University, and approved by the Ethics Committee of West China Hospital/Laboratory Animal Center.

**LNP siRNA formulation**

siRNA-loaded LNP formulations were obtained using a previously reported microfluidic rapid mixing method. The lipid mixture was dissolved in ethanol (total concentration, 13.5 mM) at a precise molar ratio, and 2′-O-methyl-modified siRNA was dissolved in 25 mM acetate buffer (pH, 4.0). The aqueous siRNA solution was rapidly mixed with the lipid mixture in a 3:1 ratio by volume (total flow rate, 2.4 mL/min) using a microfluidic mixing device (LNP-B0, FluidicLab, Shanghai, China). The SA-Pchs-LNPs solution was finally set at a DLin-MC3-DMA/1,2-distearoyl-sn-glycero-3-phosphocholine (DSPC)/cholesterol/SA-AE-AC-CH/mPEG2000CHS ratio of 40/10/39.8/10/0.2. siRNA-LNPs were subjected to microfluidic mixing (N/P = 8.0). The LNPs were rapidly diluted with RNase-free 1× PBS to ethanol content <1% and concentrated to the RNA (75 μg/mL) using Amico Ultra-15 filters (30 kDa, Milipore) and then diluted 10-fold with 1× PBS for particle size measurement using dynamic light scattering (Nikon 380). The zeta potential of the LNPs was determined by 20-fold dilution with ddH2O using a Malvern Zetasizer Nano ZS. The LNPs were concentrated five-fold for morphology observation using cryo-TEM (FEI Talos F200C). siRNA encapsulation efficiency was measured using the Ribogreen Assay (Thermo Fisher).

**dm6A CRISPR system**

The PspCas13b, guide RNA (gRNA), and non-targeting gRNA plasmids were purchased from Addgene. The PspCas13b-ALKBH5 plasmid was constructed in our lab. gRNAs were subcloned into pLKD-U6-PspCas13b-gRNA to synthesize gRNA-containing plasmids. Lentivirus was produced using the lentiviral vector packaging system in 293T cells, and the concentrated lentiviral particles were then used to infect ESCC cells.
